# Hetairos is a histology-based artificial intelligence model for predicting central nervous system tumor methylation subtypes

**DOI:** 10.1038/s43018-026-01186-3

**Published:** 2026-06-10

**Authors:** Darui Jin, Artem Shmatko, Areeba Patel, Samuel Rutz, Lukas Friedrich, Rouzbeh Banan, Ramin Rahmanzade, Philipp Sievers, Stefan Hamelmann, Daniel Schrimpf, Kirsten Göbel, Henri Bogumil, Sybren L. N. Maas, Martin Sill, Felix E. Hinz, Abigail K. Suwala, Felix Keller, Antje Habel, Gleb Rukhovich, Ferdinand Zettl, Obada T. Alhalabi, Sebastian Ille, Jannik Sehring, Daniel Amsel, Benedikt Wiestler, Pedro Piovesan Lago, Bogdana Suchorska, Olfat Ahmad, Dominik Sturm, David Reuss, Pieter Wesseling, Adelheid Wöhrer, Frank L. Heppner, Ingmar Blümcke, Claire Delbridge, Martin Jakobs, Christel Herold-Mende, Sandro M. Krieg, Wolfgang Wick, David T. W. Jones, Stefan M. Pfister, Maysa Al-Hussaini, Yanghao Hou, Felipe D’Almeida Costa, Leonille Schweizer, Luca Bertero, Till Acker, Arnault Tauziede-Espariat, Pascale Varlet, Doron Merkler, Kristof Egervari, Hildegard Dohmen, Pablo Zoroquiain, Roger Gejman, Sebastian Brandner, Xiangzhi Bai, Andreas von Deimling, Felix Sahm, Moritz Gerstung

**Affiliations:** 1 Division of AI in Oncology, German Cancer Research Center (DKFZ), Heidelberg, Germany; 2 Image Processing Center, Beihang University, Beijing, China; 3 Faculty of Biosciences, Heidelberg University, Heidelberg, Germany; 4 Department of Neuropathology, University Hospital Heidelberg, Heidelberg, Germany; 5 Clinical Cooperation Unit Neuropathology, German Cancer Consortium (DKTK) and German Cancer Research Center (DKFZ), Heidelberg, Germany; 6 Hopp Children’s Cancer Center (KiTZ), Heidelberg, Germany; 7 Faculty of Mathematics and Computer Science, Heidelberg University, Heidelberg, Germany; 8 Department of Pathology, Leiden University Medical Center, Leiden, The Netherlands; 9 Department of Pathology, Brain Tumor Center, Erasmus MC Cancer Institute, University Medical Center Rotterdam, Rotterdam, The Netherlands; 10 Division of Pediatric Neurooncology, German Cancer Research Center (DKFZ) and German Cancer Consortium (DKTK), Heidelberg, Germany; 11 Department of Neurosurgery, University Hospital Heidelberg, Heidelberg, Germany; 12 Institute of Neuropathology, Justus Liebig University Giessen, Giessen, Germany; 13 AI for Image-Guided Diagnosis and Therapy, School of Medicine and Health, Technical University of Munich, Munich, Germany; 14 Munich Center for Machine Learning (MCML), Munich, Germany; 15 AC Camargo Cancer Center, São Paulo, Brazil; 16 Division of Pediatric Glioma Research, German Cancer Research Center (DKFZ), Heidelberg, Germany; 17 Department of Pediatric Hematology and Oncology, University Hospital Heidelberg, Heidelberg, Germany; 18 Princess Máxima Center for Pediatric Oncology, Utrecht, The Netherlands; 19 Department of Pathology, Amsterdam University Medical Centers/VUmc, Amsterdam, The Netherlands; 20 Division of Neuropathology and Neurochemistry, Department of Neurology, Comprehensive Center for Clinical Neurosciences and Mental Health, Medical University of Vienna, Vienna, Austria; 21 Institute of Neuropathology and Neuromolecular Pathology, Medical University of Innbruck, Innsbruck, Austria; 22 Department of Neuropathology, Charité-Universitätsmedizin Berlin, corporate member of Freie Universität Berlin, Humboldt-Universität zu Berlin, Berlin Institute of Health, Berlin, Germany; 23 German Center for Neurodegenerative Diseases (DZNE) within the Helmholtz Association, Berlin, Germany; 24 Department of Neuropathology, University Hospital Erlangen, Friedrich-Alexander University Erlangen-Nürnberg, Erlangen, Germany; 25 Institute of Pathology, School of Medicine and Health, Technical University of Munich, Munich, Germany; 26 Division for Stereotactic Neurosurgery, Department of Neurosurgery, University Hospital Heidelberg, Heidelberg, Germany; 27 Neurology Clinic, University Hospital Heidelberg, Heidelberg, Germany; 28 Clinical Cooperation Unit Neurooncology, German Cancer Consortium (DKTK) and German Cancer Research Center (DKFZ), Heidelberg, Germany; 29 National Center for Tumor Diseases (NCT), Heidelberg, Germany; 30 Department of Cell Therapy and Applied Genomics, King Hussein Cancer Center, Amman, Jordan; 31 Department of Pathology and Laboratory Medicine, King Hussein Cancer Center, Amman, Jordan; 32 Department of Pathology, Center for Molecular Medicine Testing, College of Basic Medicine, Chongqing Medical University, Chongqing, China; 33 Center for Medical Epigenetics, School of Basic Medical Sciences, Chongqing Medical University, Chongqing, China; 34 DASA Laboratories, São Paulo, Brazil; 35 Edinger Institute, Institute of Neurology, University of Frankfurt am Main, Frankfurt am Main, Germany; 36 German Cancer Consortium (DKTK) Partner Site Frankfurt/Mainz and German Cancer Research Center (DKFZ), Heidelberg, Germany; 37 Frankfurt Cancer Institute (FCI), Frankfurt am Main, Germany; 38 Department of Medical Sciences, University of Turin, Turin, Italy; 39 Department of Neuropathology, Sainte-Anne Hospital, Paris, France; 40 Inserm, UMR 1266, IMA-Brain, Institut de Psychiatrie et Neurosciences de Paris, Paris, France; 41 Department of Pathology and Immunology, University of Geneva, Geneva, Switzerland; 42 Division of Clinical Pathology, Geneva University Hospital, Geneva, Switzerland; 43 Pathology Department, Faculty of Medicine, Pontificia Universidad Católica de Chile, Santiago, Chile; 44 Department of Neurodegenerative Disease, UCL Queen Square Institute of Neurology, London, UK

**Keywords:** CNS cancer, Cancer imaging, Cancer, Computational biology and bioinformatics

## Abstract

Molecular testing is essential for classifying central nervous system (CNS) tumors, with methylation profiling providing the highest diagnostic granularity. However, this requires more resources and time than conventional hematoxylin and eosin (H&E) histopathology, which is widely available globally. Here we propose Hetairos, an artificial intelligence algorithm that predicts 102 methylation-based CNS tumor subtypes from digital H&E slides. Built and validated on 9,606 patients and over 11,000 slides from 11 centers across four continents, Hetairos identified 50–70% of cases with high confidence, achieving an accuracy of 0.87 for its highest-rated predictions. Hetairos outperformed five board-certified neuropathologists in a direct histology-only comparison (0.68 versus 0.30). Prospective evaluation in routine diagnostics confirmed its performance, reducing turnaround time from 12 days (molecular testing) to 12 min. Hetairos supports diagnostic decision-making across the full spectrum of pediatric and adult CNS tumors by narrowing differential diagnoses and guiding efficient testing.

## Abstract (web summary)

Jin et al. developed an artificial intelligence tool that leverages histology to predict methylation-based central nervous system tumor subtypes and validated it for tumor classification, demonstrating high confidence and accuracy across clinical cohorts.

## Main

Central nervous system (CNS) tumors encompass a wide range of neoplasms characterized by morphological and molecular heterogeneity. The diagnosis of these tumors has been profoundly changed by various molecular tests, in particular DNA methylation profiling, which has helped establish a granular molecular taxonomy^1^. Methylation-based tumor subtyping has been shown to stratify outcomes more accurately than previously established classification systems, leading to its widespread adoption^2–6^. As a result, the 2021 World Health Organization (WHO) classification of CNS tumors, which distinguishes 108 tumor subtypes, has now incorporated class definitions derived from methylation profiling^7^. For most tumor types and subtypes, methylation profiling is recommended as a desirable diagnostic test and is indispensable for achieving certain diagnoses—such as high-grade astrocytoma with piloid features (HGAP) and diffuse glioneuronal tumors with oligodendroglioma-like features and nuclear clusters (DGONC)—as well as for the molecular subtyping of medulloblastomas or ependymomas.

Despite the successes of methylation-based classification, there are drawbacks to this approach. Firstly, it requires a considerable investment in equipment, making it inaccessible to a substantial portion of the global population^8^. Furthermore, methylation analysis requires large amounts of tumor material, which can be limiting in the case of stereotactic biopsies^9^. Finally, DNA methylation analysis has a typical turnaround time of 2 weeks, which can delay a definitive diagnosis^10^. Faster and more widely accessible methods are therefore needed. While nanopore sequencing shows great potential, it also requires bespoke instruments and fresh or fresh-frozen tissues^11–13^. Currently, routine diagnosis is still largely based on histopathological assessment of hematoxylin and eosin (H&E)-stained tissue sections—typically the first step in tissue-based diagnostics before molecular testing^14^. While histopathological analysis is not sufficient to definitively diagnose the full spectrum of CNS tumors, H&E tissue sections are widely and readily available.

Recent advances in computer vision have led to notable successes in digital pathology. Algorithms have been shown to detect morphological features of molecular alterations and to classify tumors into molecularly defined categories^15–20^. A series of recent foundation models, trained on hundreds of thousands of histopathology slides to recognize diverse histopathological features, provide the basis for many current digital pathology models^21–24^. These advances may be particularly beneficial for diagnosing CNS tumors, which are difficult to identify using conventional histopathological approaches. A recent analysis showed that methylation signals could be estimated from standard diagnostic H&E sections of formalin-fixed, paraffin-embedded (FFPE) tissue, providing a means to diagnose ten different tumor types^25^. Other studies have demonstrated the possibility of predicting specific molecular alterations^26,27^ and classifying distinct subtypes within diffuse gliomas^28^ and spinal cord ependymomas^29^. However, an artificial intelligence (AI)-based diagnostic solution for H&E slides that covers the entire spectrum of CNS tumors, as currently only possible with methylation testing, is still missing.

Here, we describe Hetairos, an AI-based model that classifies whole-slide images of H&E-stained FFPE tissue sections into 102 subtypes of CNS tumors. Hetairos was trained and evaluated on 9,606 tumors, comprising more than 11,000 slides from 11 different institutions across four continents. Hetairos provided high-confidence predictions on 50–70% of the slides, with an accuracy of 0.87. The algorithm outperformed human neuropathologists in a side-by-side assessment of 210 cases selected from the full spectrum of CNS tumors. Lastly, we prospectively evaluated Hetairos in a routine diagnostic setting involving 210 tumor samples. Our results suggest that AI-based predictions can lead to faster diagnoses and help resolve cases with ambiguous results in methylation analysis or insufficient genomic material.

## Results

### Hetairos is trained to predict 102 tumor subtypes

**Fig. 1:**
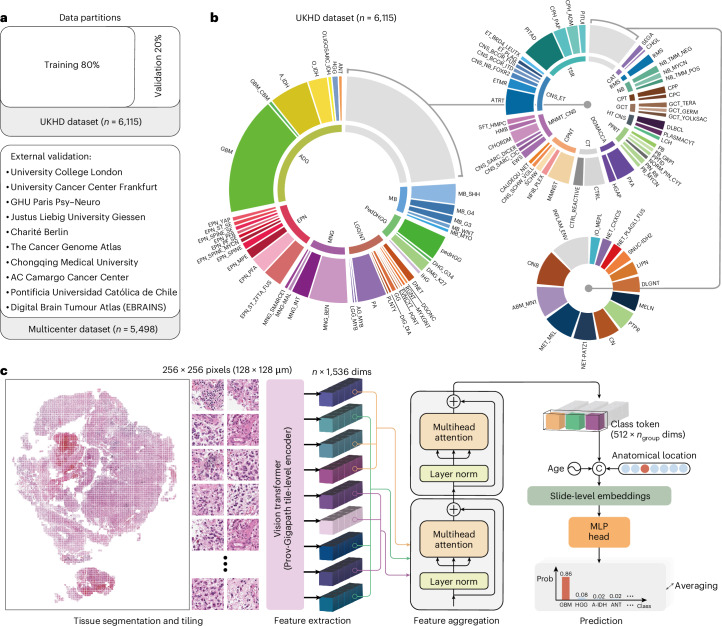
Overview of study cohorts and Hetairos’s architecture. **a**, Summary of the internal dataset and the ten external datasets used in this study. Hetairos was trained and evaluated using the UKHD dataset (*n* = 6,115 slides). The trained model was additionally evaluated on a multicenter cohort to ensure generalizability (*n* = 5,498 slides). **b**, Tumor subtype distribution for the UKHD dataset (*n* = 6,115 slides). The outer ring displays the 102 tumor subtypes, while the inner ring denotes their corresponding supertypes. Full names and abbreviations of tumors are listed in Supplementary Table 1. **c**, Schematic of slide processing by Hetairos. Whole-slide images are split into nonoverlapping tiles, from which features are extracted using a pretrained vision transformer. Tile features, along with age and tumor location, are combined into a slide-level representation. Finally, an MLP predicts probabilities for each of the 102 tumor subtypes from the slide-level representation. dims, dimensions. Source data

Hetairos was built using 6,115 slides from 4,961 patients with CNS tumors at the Department of Neuropathology, University Hospital Heidelberg (UKHD). All cases were annotated with molecular data to comply with or exceed the requirements of the 2021 WHO classification (CNS5+). This cohort includes CNS tumors from all age groups and is intended to largely mirror the incidence of tumor subtypes, with deliberate enrichment of some rare tumor types. Twenty percent of the UKHD dataset was used for internal validation. Hetairos was subsequently validated on ten external cohorts from four continents, comprising an additional 4,645 tumors and 5,498 slides (Fig. 1a). For each tumor, one or multiple slides containing H&E-stained tissue sections scanned at a magnification of at least 20× (approximately 0.5 µm per pixel) were available, together with the paired methylation-based molecular classification (Methods).

Methylation classifications were generated using version 12.8 of the Molecular Neuropathology methylation classifier. The predicted classes were further grouped—incorporating expert neuropathological input—into a simplified classification system of 102 diagnostically relevant tumor subtypes and 34 superfamilies, covering the full spectrum of CNS tumor subtypes (Methods, Fig. 1b and Supplementary Table 1). These 102 subtypes consolidate a range of provisional tumor subtypes from the full set of 184 methylation-based classes, which are either very rare or whose clinical relevance has yet to be established. The aggregated methylation-based subtypes were used as the ground truth for training Hetairos.

In line with epidemiology, the distribution of tumor classes in the UKHD dataset followed a long-tailed pattern, with around 30% of cases belonging to the superfamily of adult-type diffuse gliomas, including isocitrate dehydrogenase (IDH)-wild-type glioblastoma (Fig. 1b). Another 50% of cases originated from the superfamilies of ependymomas, meningiomas, low-grade glial/glioneuronal tumors, pediatric high-grade gliomas, and medulloblastomas.

The Hetairos model predicted each of the 102 CNS tumor subtypes from H&E whole-slide scans (Fig. 1c). Because the scanned slides were too large to be processed in a single step, they were first tiled into nonoverlapping areas of approximately 128 × 128 µm, followed by computational feature extraction using the Prov-GigaPath vision transformer model^22^. The resulting 1,536-dimensional features per tile were aggregated into slide-level embeddings to predict each of the 102 CNS tumor subtypes using a modified TransMIL model^30^ (Methods). This aggregation step enabled Hetairos to learn which image tiles are predictive and to focus on the most informative image areas. Additionally, patient age and anatomical tumor location can be incorporated to enhance model performance (see the ablation results in Supplementary Table 2). The model outputs the probability of each of the 102 tumor subtypes. By considering only local subsets of tiles, Hetairos can generate prediction maps that highlight the image areas most indicative of given tumor classes. More details are provided in the Methods.

### Accurate classification of CNS tumors and identification of ambiguous cases

**Fig. 2:**
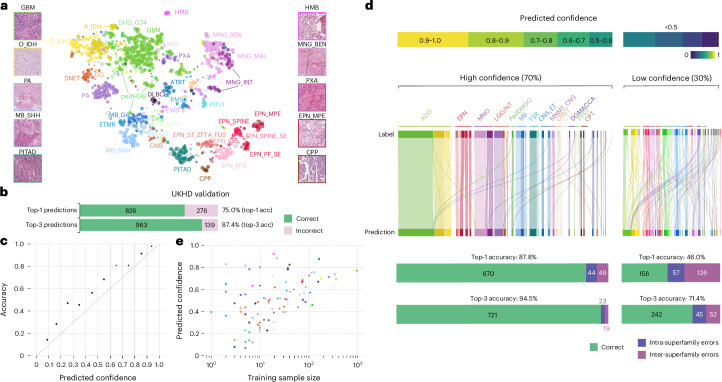
Performance of Hetairos in internal validation (*n* = 1,102 slides). **a**, Neighborhood embedding of slide representations. Slide-level embeddings from the internal validation set are visualized in two dimensions using UMAP (number of neighbors = 5, minimum distance = 0.5). Representative tiles for clusters are shown on either side. **b**, Accuracy of the most probable (top-1) and three most probable (top-3) subtypes predicted by Hetairos. **c**, Scatter plot illustrating the calibration of Hetairos’s predicted probabilities. **d**, Hetairos’s predictive performance across different confidence intervals. **e**, Scatter plot showing the correlation between training sample size and Hetairos’s predicted confidence. Source data

Over the past decade, the discovery of new tumor classes has been primarily based on molecular analyses, including methylation profiling. This approach consistently reveals clusters of tumors with shared underlying biological relationships that often appear morphologically unrelated. Intriguingly, a visualization of slide-level embeddings for the internal validation cohort using UMAP^31^ revealed that Hetairos learned internal representations that similarly cluster different groups of tumors (Fig. 2a). While the clustering is not yet as distinct as that found by methylation analysis, the emerging structures reflect the histopathological similarity of different tumor superfamilies, such as glial tumors, ependymal tumors, meningiomas and medulloblastomas.

The visible clustering of subtypes also reflects Hetairos’s ability to distinguish different tumor classes. Hetairos assigns a probability to each possible class, which usually centers around a single class or a few related classes. Of greatest interest is typically the class with the highest probability, which we term the top-1 prediction, and the corresponding estimated probability, which we refer to as Hetairos’s confidence. Top-1 predictions agreed with the methylation classification results (methylation score > 0.8) in 75% of all internal validation tumors (Fig. 2b) and remained robust in the presence of common histological artifacts (Supplementary Tables 3 and 4); additional performance metrics are provided in Supplementary Table 5. In 87% of cases, the true class label was among Hetairos’s three most likely classes (top-3 accuracy). Reassuringly, incorrect predictions were typically assigned lower confidence scores, showing a conservative tendency that is important to avoid confident mispredictions (Fig. 2c). In line with the methylation classifier, it is important for users of Hetairos to know the confidence levels at which the predictions are typically correct. We designated cases with confidence above 0.5 as ‘high confidence’ and those with confidence below 0.5 as ‘low confidence’. High- and low-confidence cases comprised 70% and 30% of tumors in the internal validation cohort, respectively (Fig. 2d). Hetairos’s top-1 accuracy among the high-confidence set was 0.88, demonstrating that the model can deliver an accurate and detailed initial diagnosis in the majority of cases, with accuracy further increasing as the confidence threshold rises (Supplementary Table 6).

In the low-confidence set, the accuracy dropped, as expected, to 0.46. When combining the three most likely predictions for the low-confidence set, the accuracy was still 0.71, which shows that Hetairos can often meaningfully reduce the set of possible diagnoses from 102 subtypes to just 3 even in low-confidence cases. Such narrowing of probable classes may help guide further diagnostic tests, potentially resolving the differential diagnoses with only a few or even a single immunohistochemistry test, single gene assay or chromosomal hybridization, rather than through high-throughput testing.

**Fig. 3:**
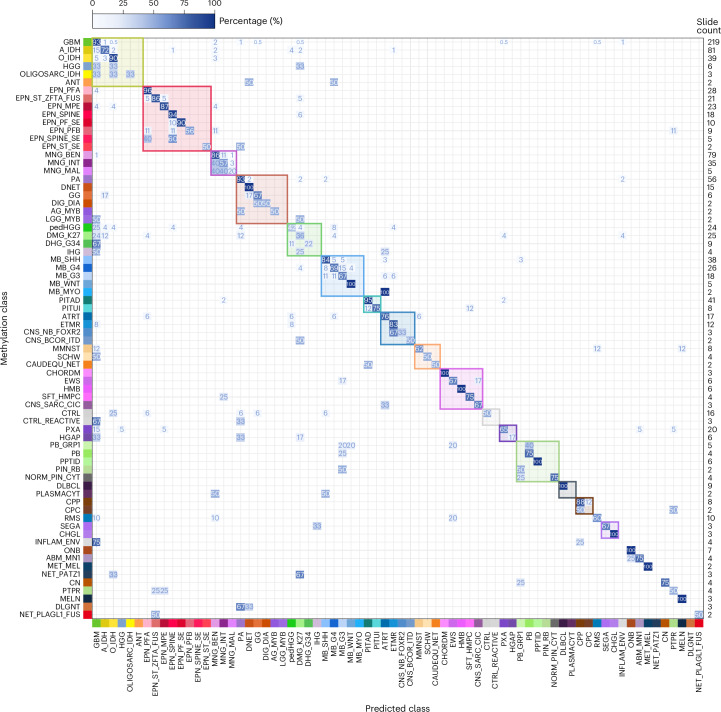
Confusion matrix for internal validation by Hetairos (*n* = 1,102 slides). The matrix provides a comprehensive view of classification accuracy across 72 CNS tumor subtypes (classes with a sample size >1), with the vertical axis representing the true classes and the horizontal axis representing the predicted classes. Each cell displays the percentage of cases in each actual class that are classified into each predicted class. Rectangle boxes group classes that belong to the same superfamily. The sample size of each class is listed on the right. Source data

**Extended Data Fig. 1:**
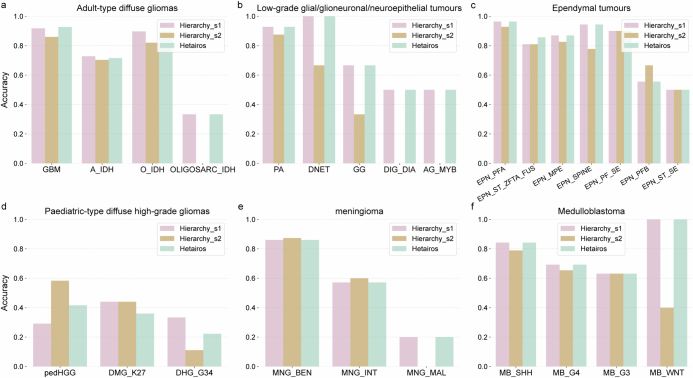
Class-wise accuracy comparison between hierarchical models and Hetairos within different tumour superfamilies. **a-f**, class-wise accuracy within the families of adult-type diffuse gliomas, low-grade glial/glioneuronal/neuroepithelial tumours, ependymal tumours, paediatric-type diffuse high-grade gliomas, meningioma and medulloblastoma. **Hierarchy s1**: We first applied the Hetairos to predict the subtype probabilities for each sample. Based on the predefined mapping between subtypes and superfamilies, we inferred the corresponding predicted superfamily. Within this predicted superfamily, the final subtype was determined as the class with the highest probability. **Hierarchy s2**: Using the same hyperparameters as the original Hetairos, we first trained a model to classify 34 tumor superfamilies. Subsequently, separate subtype classification models were trained and validated within each individual superfamily. Source data

**Extended Data Fig. 2:**
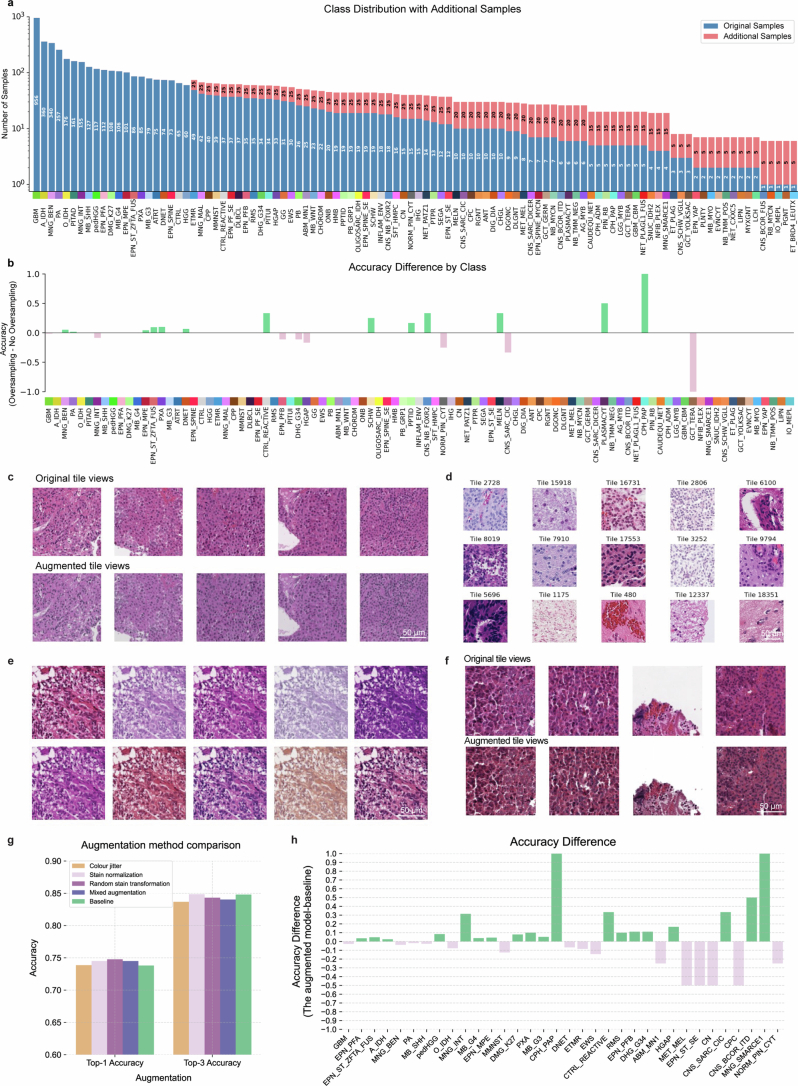
Oversampling and color space augmentation. **a**, Number of added samples per class for oversampling. The number of added samples to each class is proportional to the original sample size. **b**, Difference in per-class accuracy between models trained with and without oversampling. Classes on the x-axis are arranged in descending order of the training sample size. **c**, Examples of color jittering augmentation applied to tiles. Each slide in the training set was augmented using random transformations. **d**, Representative tiles used as reference for stain normalization. **e**, Difference versions of normalized tile views. The Macenko Method is applied in the stain normalization. **f**, Examples of random stain transformation applied to tiles. Random perturbations were introduced to the concentration parameters to get the augmented views. **g**, Top-1 and top-3 accuracy on the internal validation for different augmented models. Baseline: the model trained on the dataset without any augmentations. **h**, Difference in class-wise accuracy between models trained with color space augmentations and the baseline model. Source data

For half of the errors Hetairos made on the high-confidence cases, the correct class belonged to the same superfamily as the predicted class, resulting in an accuracy of 0.94 at the superfamily level. This pattern reflects the close morphological similarity of these entities and their consistent confusion, even within the hierarchical classification systems (Extended Data Fig. 1a–f). Among the low-confidence set, errors tended to occur across superfamilies and subtypes. In such errors, classes with higher incidence in the training data were predicted more often and with greater confidence. All 12 tumor subtypes with an average confidence below 0.25 had fewer than 20 occurrences in the training set (Figs. 2d,e and 3). Data augmentation strategies, including oversampling and color space transformation, appeared insufficient to further improve performance on these underrepresented classes (Extended Data Fig. 2a–h). These findings highlight the need for large datasets to confidently classify very rare tumor subtypes, such as liponeurocytomas, atypical teratoid/rhabdoid tumors and germinomas.

### Hetairos’s accuracy is preserved in external validation cohorts

**Fig. 4:**
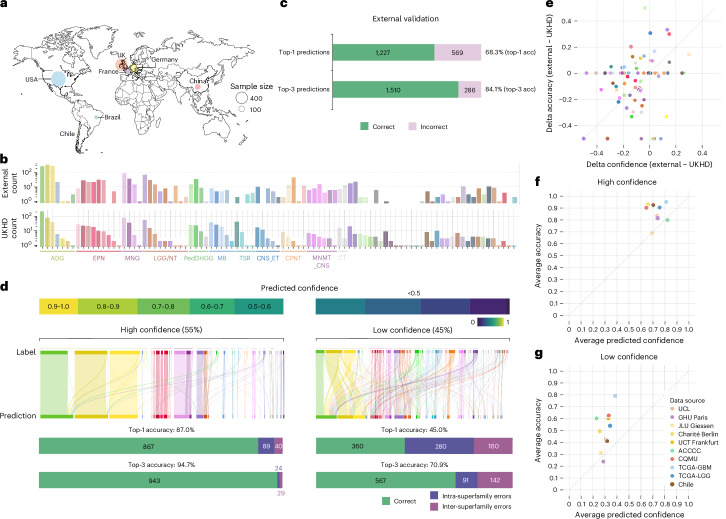
Performance of Hetairos in external validation (*n* = 1,796 slides). **a**, External validation data consist of nine cohorts from seven countries across four continents. **b**, Bar plots showing the tumor subtype distribution in the external validation set. The observed frequencies closely mirror those of the internal validation set. **c**, Prediction accuracy on the external validation set. Hetairos achieved a top-1 accuracy of 0.68 and a top-3 accuracy of 0.84 in external validation. The green bar denotes the correct predictions, and the pink bar denotes the incorrect ones. **d**, Hetairos’s predictive performance across different confidence intervals in external validation. **e**, Scatter plot illustrating the correlation between differences in confidence and differences in accuracy between internal and external validation. Here, the differences were calculated as the values from the external validation set minus those from the internal validation set. **f**,**g**, Scatter plots illustrating the correlation between average predicted confidence and accuracy across different cohorts, grouped into high-confidence predictions (**f**) and low-confidence predictions (**g**). UCL, University College London; GHU Paris, Groupe Hospitalier Universitaire Paris Psychiatrie & Neurosciences; JLU Giessen, Justus Liebig University Giessen; UCT Frankfurt, University Cancer Center Frankfurt; ACCCC, AC Camargo Cancer Center; CQMU, Chongqing Medical University; Chile, Pontificia Universidad Católica de Chile. Source data

To be widely applicable, digital pathology models must maintain predictive performance across centers, demographics and processing protocols^32,33^. To evaluate Hetairos’s performance in settings unobserved during training, we assembled ten validation cohorts from different institutions across four continents, comprising 4,645 cases and 5,498 slides covering the incident spectrum of CNS tumor diagnoses (Fig. 4a,b).

**Extended Data Fig. 3:**
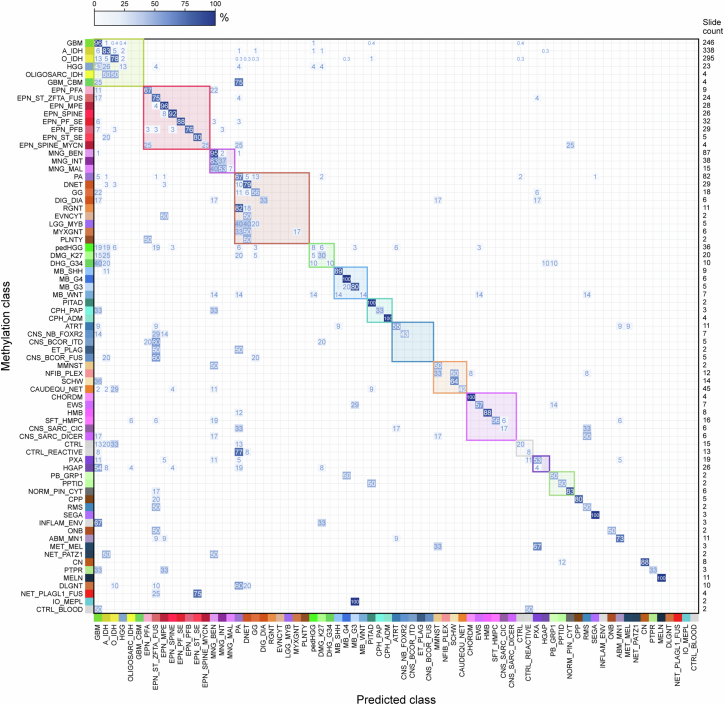
Confusion matrix for external validation by Hetairos. The matrix provides a comprehensive view of classification accuracy across 72 CNS tumour subtypes (classes with sample size > 1), with the vertical axis representing the methylation classes and the horizontal axis the predicted classes. Each cell displays the percentage of cases in each methylation class classified as each predicted class. Rectangle boxes group classes that belong to the same superfamily. The sample size of each class is listed on the right side. Source data

**Extended Data Fig. 4:**
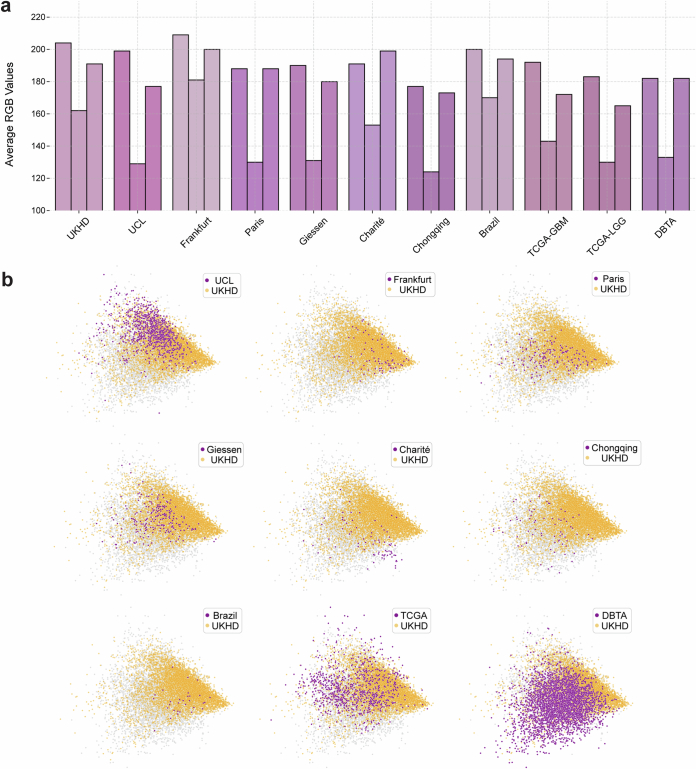
Color space distribution of different data centers. **a**, Average R/G/B values for samples from different centers. Each center is represented by three bars from left to right, corresponding to the average values of the R, G, and B channels, respectively. The color of each bar reflects the RGB values of the respective center. **b**, PCA-based visualization of color embeddings from different centers. Each dot represents an H&E slide image. Gray points represent all external data points that are not from UKHD or the highlighted center. Source data

Hetairos’s overall accuracy was lower in external validation cohorts than in the internal validation cohort (68% versus 75%) (Fig. 4c, Extended Data Fig. 3 and Supplementary Table 5). Reassuringly, however, this discrepancy was largely predicted, as the fraction of low-confidence predictions increased from 30% to 45% (Fig. 4d). This discrepancy may in part be attributed to modality differences between the external and internal validation sets (Extended Data Fig. 4a,b and Supplementary Table 7). Among the 55% high-confidence cases, the top-1 accuracy remained 0.87 (0.96 at the superfamily level), similar to the performance achieved in the internal validation cohort. Among low-confidence cases, the prediction accuracies were 0.45 for top-1 and 0.71 for combined top-3 predictions, consistent with those of low-confidence samples in the internal validation cohort. This provides evidence that Hetairos recognizes differences in slide characteristics and adjusts its confidence levels accordingly.

The change in average confidence per tumor class was −0.10 (range −0.53 to +0.30) and was usually accompanied by a corresponding change in observed accuracy, thereby maintaining calibration (Fig. 4e). While 59 of 79 tumor types exhibited a drop in confidence, there was also an increase in confidence and accuracy for 8 of the 79 tumor types, including medulloblastoma group 3 and schwannoma.

The external cohorts have distinct tumor class compositions due to the specialization of the individual centers. Nonetheless, the drop in confidence was of a similar magnitude across subcohorts. The accuracy on the respective high- and low-confidence cases matched or exceeded the model’s confidence, indicating that Hetairos’s predictions remain conservative (Fig. 4f,g).

**Extended Data Fig. 5:**
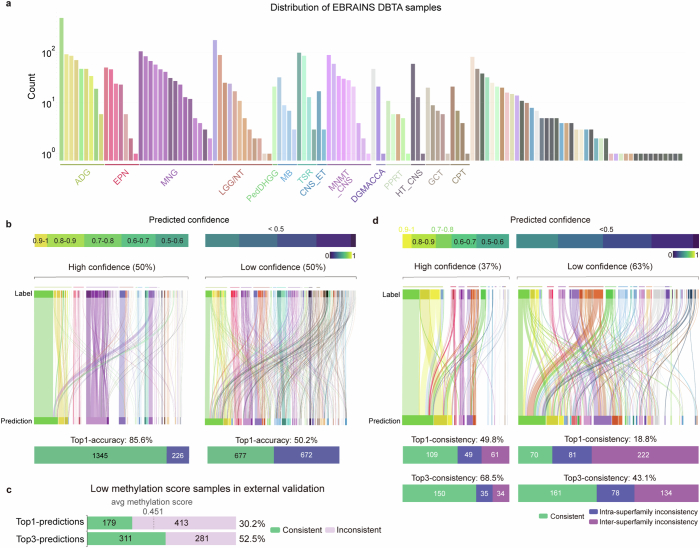
Performance of Hetairos validated on EBRAINS DBTA data (*n* = 3,110 slides) and samples with low methylation scores (<0.8, *n* = 584 slides) from the external centers. **a**, Barplots showing the tumour subtype distribution of EBRAINS DBTA dataset, labelled according to the original class definitions of the dataset. **b**, Hetairos’s predictive performance across different confidence intervals on EBRAINS DBTA data. The top row shows the distribution of prediction confidence, with high-confidence cases (50%) on the left and low-confidence cases (50%) on the right. Below, Sankey diagrams illustrate tumour classifications, approximately grouped and colour-coded by major tumour families despite different class definitions between EBRAINS DBTA and those predicted by Hetairos. The top-1 accuracy of high-confidence predictions and low confidence predictions are 85.6% and 50.2% respectively, calculated based on an expert evaluation of whether the predicted classes were consistent with the EBRAINS annotations. 190 samples in the low-confidence group were excluded from the accuracy calculation as their true classes were not included in Hetairos’ label set. **c**, Hetairos top-1 and top-3 prediction concordance with the corresponding predictions from the methylation classifier. **d**, Hetairos’s predictive performance across different confidence intervals compared to predictions from the methylation classifier. Source data

Lastly, Hetairos was evaluated qualitatively on the EBRAINS Digital Brain Tumour Atlas (DBTA) cohort, which comprises 3,110 slides lacking methylation-based predictions. Instead, Hetairos predictions were compared to the detailed histopathological diagnoses provided (Extended Data Fig. 5a,b). Specific histology class names and their corresponding color codes are detailed in Supplementary Table 8. In this cohort, Hetairos’s confidence distribution was similar to that in other external validation cohorts, showing a 50–50% split between high- and low-confidence cases, with corresponding accuracies of 85.6% and 50.2%, respectively. The predicted tumor types showed good agreement with histopathological diagnoses, with discrepancies mostly occurring within tumor families. Additional results on external samples with low methylation scores (<0.8, with an average of 0.45) are provided in Extended Data Fig. 5c,d (note that methylation class annotations may differ from the final diagnoses for these cases).

### Hetairos outperforms neuropathologists in H&E assessment

**Fig. 5:**
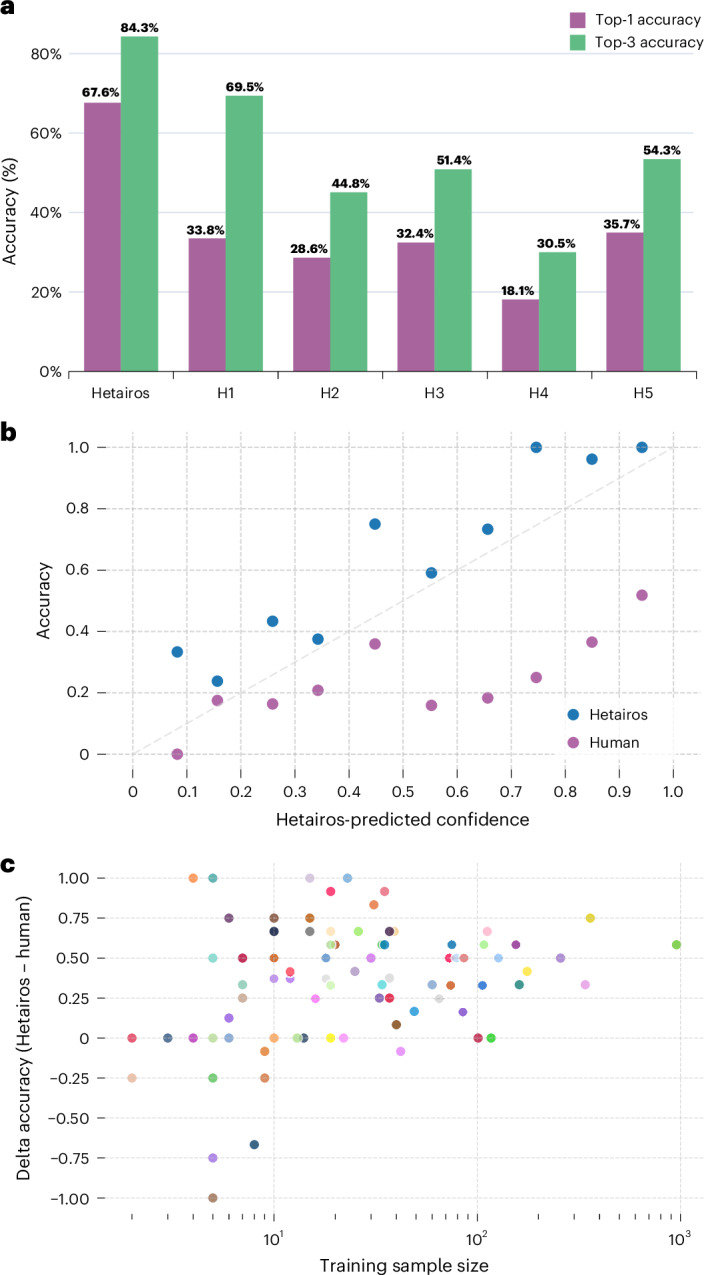
Hetairos outperforms neuropathologists in comparative testing (*n* = 210 slides). **a**, Bar plot comparing the top-1 and top-3 accuracies achieved by Hetairos and five neuropathologists (H1–H5) in the side-by-side comparison. Purple bars represent top-1 accuracy, while green bars represent top-3 accuracy. **b**, Scatter plot comparing the prediction accuracy of Hetairos and neuropathologists across different confidence intervals. Blue dots represent Hetairos’s accuracy, while purple dots represent the average accuracy of neuropathologists. The intervals were defined by Hetairos’s confidence. **c**, Scatter plot illustrating the correlation between training sample size and the accuracy difference between Hetairos and neuropathologists across tumor subtypes (represented by differently colored dots). Source data

Identifying and narrowing differential diagnoses from H&E slides is an important first step in the diagnostic workflow and is pivotal for the efficient selection of subsequent diagnostic tests. While many institutes in high-income countries typically have a set of special stains (for example, PAS and reticulin) and immunohistochemistry tests (for example, GFAP, MAP2, NeuN and synaptophysin) readily available at initial inspection to assess tumor lineage, such diagnostic tools are often absent or restricted to a few cases in settings with more limited resources. Hence, we conducted a blinded side-by-side evaluation of 210 slides by five board-certified neuropathologists and Hetairos. The slides were selected to have a similar number of cases from each class (Supplementary Table 9). Neuropathologists were provided with a drop-down list containing the 102 methylation subtypes, which correspond directly to Hetairos’s output classes, and were asked to choose and rank their top-3 diagnoses from it. The neuropathologists participating in the evaluation had prior experience with methylation classification and were thus familiar with the provided classes. Based on H&E-stained sections only, Hetairos’s accuracy was consistently better than that of neuropathologists, who achieved an average top-1 accuracy of 0.30 (0.18–0.36) compared to Hetairos’s 0.68 (see the additional metrics provided in Supplementary Table 5). This gap narrowed when assessing top-3 accuracy, which was 0.50 (0.31–0.70) for humans and 0.84 for Hetairos (Fig. 5a). Human evaluators often appeared to struggle with identifying the single best choice among a large number of granular classes, but they were able to provide a plausible set of diagnoses. While Hetairos provided a calibrated range of confidence levels, it consistently outperformed human accuracy across all confidence intervals. The performance gap between human evaluators and Hetairos narrowed slightly within Hetairos’s lower confidence range (Fig. 5b).

When considering individual tumor subtypes, Hetairos generally outperformed humans for classes represented by more than ten cases in the training cohort (Fig. 5c). For rare tumor subtypes with fewer than ten cases in the training cohort, human pathologists performed similarly to Hetairos. For some diagnoses, such as metastatic melanoma and teratoma—which Hetairos struggled to diagnose—human diagnoses were correct in two out of three cases and one out of one case, respectively. Together, these results indicate that Hetairos is currently better at diagnosing all but the rarest types of tumors.

### AI-assisted diagnosis reaches methylation-level accuracy in 12 min

**Fig. 6:**
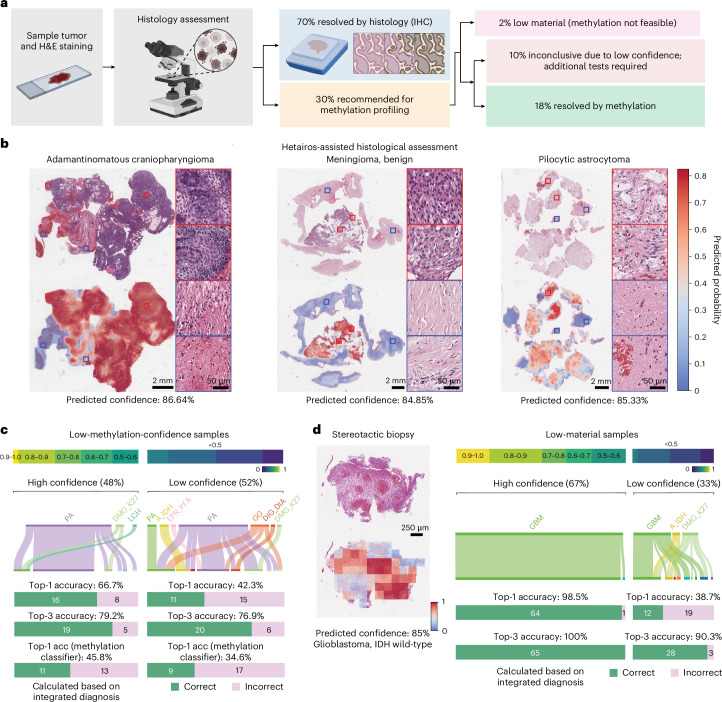
Hetairos assists in the pathological evaluation and resolution of molecularly inconclusive cases. **a**, Flowchart of diagnostic steps for CNS tumors in neuropathology. Following histological assessment of H&E slides, approximately 30% of the samples require further molecular testing. Among these, 2% are low-material samples and 10% are low-methylation-confidence samples for which diagnosis cannot be resolved using DNA methylation analysis. IHC, immunohistochemistry. **b**, Examples of H&E-stained sections and heatmaps corresponding to Hetairos’s highest probability prediction (shown below each image). Red-colored regions indicate areas that Hetairos identified as strongly indicative of the listed tumor class. **c**, Hetairos’s predictive performance across different confidence intervals on low-methylation-confidence samples (*n* = 50 slides). **d**, Hetairos’s predictive performance across different confidence intervals on low-material samples (*n* = 96 slides), with a stereotactic biopsy example shown on the left. Flowchart in **a** created in BioRender; Patel, A. https://biorender.com/crmoijd (2026). Source data

Named after the Greek term for ‘companion’, Hetairos is designed to assist neuropathologists in diagnostic work. As mentioned previously, the typical diagnostic workflow in neuropathology begins with a morphological assessment of an H&E section, followed by a series of immunohistochemical tests selected to narrow the differential diagnoses (Fig. 6a). Approximately 30% of cases cannot be resolved in terms of tumor classification and subtyping without advanced molecular testing. Most of these can be resolved by DNA methylation array analysis, whereas some require additional testing, such as DNA and RNA sequencing, to identify pathognomonic mutations or fusions. Some specimens are unsuitable for molecular analysis because of limited sample quantity or quality. Hetairos fits into this workflow as a tool to supplement the first-line method—that is, manual histopathological evaluation using H&E-stained FFPE slides. Owing to Hetairos’s WHO 2021-compatible granularity and well-calibrated predictions, it is intended to be used as a triaging tool to efficiently guide further molecular analyses.

**Extended Data Fig. 6:**
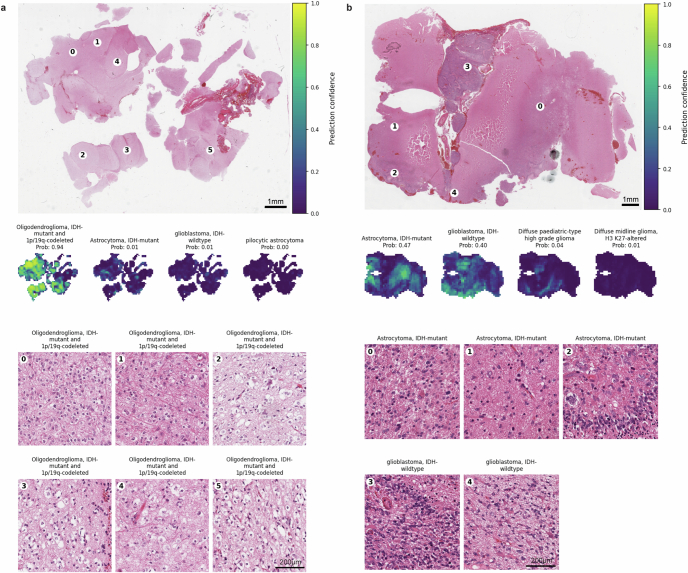
Hetairos report examples of Oligodendroglioma and astrocytoma. **a**, An example report of oligodendroglioma, IDH-mutant and 1p/19q-codeleted with high Hetairos’s confidence and typical tumour patterns. **b**, An example report of astrocytoma, IDH-mutant with a Hetairos’s confidence of 0.47, and glioblastoma, IDH-wildtype with a confidence of 0.40. Typical tumour patterns are shown at the bottom of the figure.

The report provided by Hetairos highlights tissue areas of the H&E slide that are most informative for diagnostic prediction and those that contribute least, both in a prediction map and with exemplary magnifications (Fig. 6b; see also the example reports in Extended Data Fig. 6 and Supplementary Table 10). These illustrations enable neuropathologists to review Hetairos’s decision-making and may also guide the selection of optimal areas for extraction if further molecular testing is needed. For example, areas that are morphologically informative for the diagnosis of craniopharyngioma or meningioma are robustly separated from adjacent connective tissue.

**Extended Data Fig. 7:**
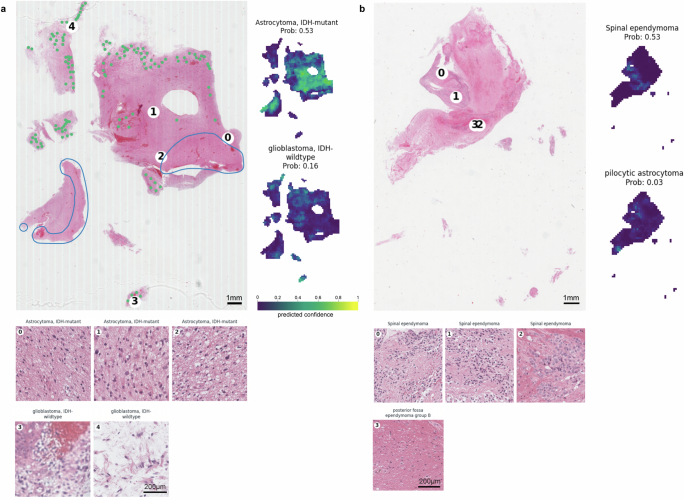
Hetairos report examples illustrating intra-tumour histological heterogeneity. **a**, An example report of astrocytoma, IDH-mutant. Blue: CNS infiltration zone; Green: spots of vascular proliferations, which are relevant criteria for assigning astrocytoma as grade 4 and therefore received higher scores in the heatmap for glioblastoma. Heatmaps of different predicted tumour classes are listed below in descending order of confidence. **b**, An example report of spinal ependymoma. The heatmap clearly delineates the relevant areas of the spinal ependymoma. The dark blue areas show features resembling those of some pilocytic astrocytomas, which are correctly de-emphasized by Hetairos in this case. Heatmaps of different predicted tumour classes are listed below in descending order of confidence.

Within the spectrum of glial morphology, pilocytic astrocytoma bulk tissue was clearly distinguished from the surrounding reactive gliosis (Fig. 6b). Similarly, Hetairos uniformly identified the prototypical histology of oligodendroglioma (Extended Data Fig. 6a). In astrocytoma, areas considered by Hetairos to be indicative of the second-best diagnosis, glioblastoma, coincided with microvascular proliferation—a characteristic feature of both diagnoses and a criterion for grade 4 tumors (Extended Data Figs. 6b and 7a,b).

**Extended Data Fig. 8:**
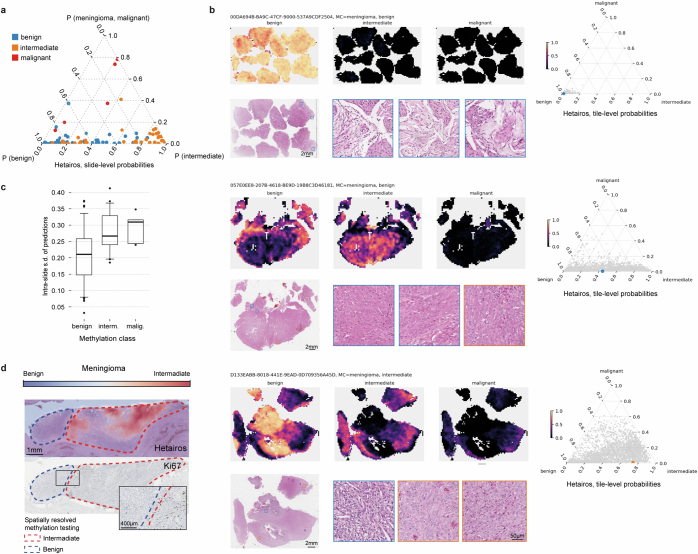
Hetairos identifies heterogeneity within meningioma subtypes. **a**, Triplot showing the predicted probabilities for slides from three meningioma subtypes. **b**, Examples showcasing the predictive heatmaps generated by Hetairos for three meningioma cases, illustrating intra-tumour heterogeneity. Representative benign/intermediate patterns are shown below. **c**, Distribution of intra-slide prediction variability for the three meningioma subtypes (meningioma benign: *n* = 79 slides; intermediate: *n* = 35 slides; malignant: *n* = 5 slides), showing that low heterogeneity only occurs in benign cases. Box plots show the median (centre line), 25th–75th percentile (box bounds), 5th–95th percentile (whiskers). **d**, Paired H&E and IHC samples were used to validate the intratumoral heterogeneity predicted by Hetairos. The highlighted regions in the heatmaps show high concordance with the Ki67-stained areas. Source data

Further examples of intratumoral heterogeneity were observed in meningiomas, where Hetairos frequently identified regions assigned to the benign category within intermediate-group cases and vice versa (Extended Data Fig. 8a–d). The biological underpinning of the distinct grading classes found within the same tumor was supported by Ki-67 staining patterns (that is, weaker staining in areas predicted to be benign). Taken together, these examples illustrate that Hetairos has the potential to capture subtle yet clinically relevant and biologically meaningful histologies that exhibit intratumoral heterogeneity and pose diagnostic challenges.

**Extended Data Fig. 9:**
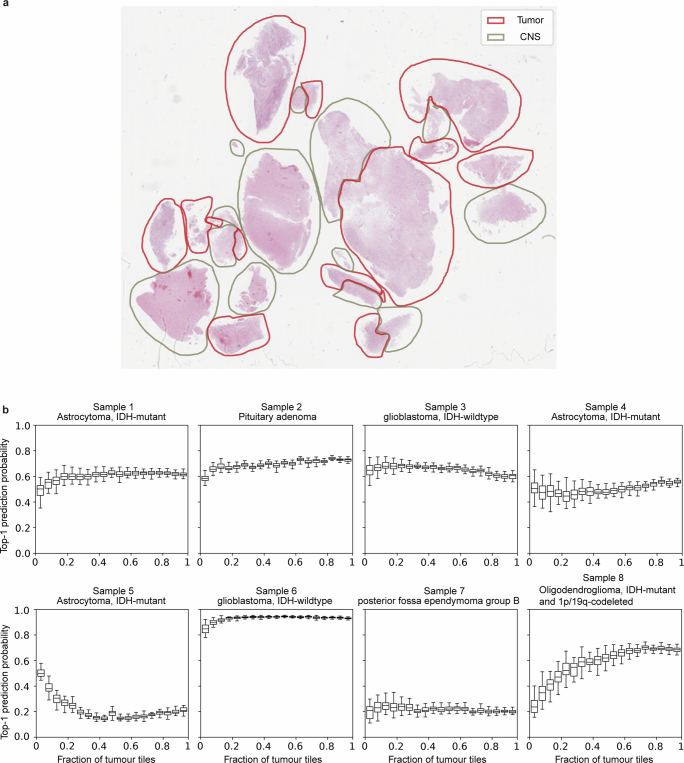
Hetairos is robust to intratumoral heterogeneity. **a**, Example annotation of a slide to tumour/CNS (healthy) tissue. **b**, Hetairos top-1 prediction confidence across subsamples (*n* = 50 subsets within one slide) with varying fractions of tumour tissue. Box plots show the median (centre line), 25th–75th percentile (box bounds), 1.5× IQR (whiskers). Source data

Even advanced technologies such as methylation analysis may sometimes fail to provide a clear prediction on their own, thus necessitating additional testing (for example, for fusions or mutations). Within a cohort of 50 samples diagnosed solely based on a combination of molecular assays, Hetairos correctly predicted 27 cases, demonstrating superiority over methylation analysis in some scenarios (Fig. 6c). For 96 specimens in which methylation analysis could not be performed because of limited tissue, particularly stereotactic biopsy samples, Hetairos correctly predicted 76 cases (Fig. 6d). This highlights the robustness and predictive capability of Hetairos in specimens with limited tumor content (Extended Data Fig. 9a,b).

**Fig. 7:**
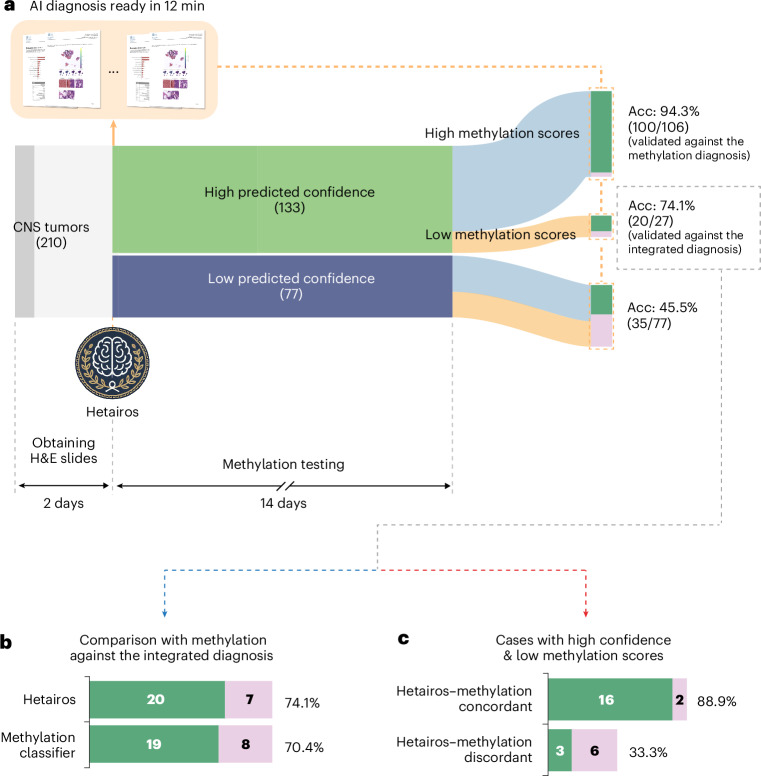
Prospective clinical evaluation of Hetairos (*n* = 210 slides). **a**, Sankey plot showing the diagnostic path for the prospective validation cohort and the performance of Hetairos on those samples. Obtaining Hetairos’s predictions takes an average of 12 min once the scanned H&E slide is available. **b**, Comparison between the results from Hetairos and those from the methylation classifier on prospective samples with low methylation scores. **c**, Classification accuracy in low-methylation-score cases concordant or discordant with Hetairos’s high-confidence predictions.

To assess the potential clinical utility of Hetairos, the algorithm was prospectively evaluated alongside routine diagnostics from August 1, 2024, to June 1, 2025, at the Department of Neuropathology at UKHD. All cases that required molecular testing and met the inclusion criteria were included without any further selection. During this time, Hetairos was used to predict a total of 210 cases (Fig. 7a and Supplementary Table 11). Its results were compared to independent integrated diagnoses established by a combination of morphological assessment, immunohistochemistry, methylation classification, next-generation sequencing panel and RNA sequencing. Hetairos predictions were not made available to the neuropathologist and did not influence diagnostic or treatment decisions.

On average, it takes 12 days from the receipt of the neurosurgical specimen to an integrated diagnosis. As illustrated in Fig. 7a, this corresponds to approximately 16 days for cases requiring molecular testing. Hetairos, running on consumer-grade hardware, took an average of 12 min to process a slide and generate the report. This indicates that, together with the time taken for staining and scanning, Hetairos substantially shortens the turnaround time, with results usually available within 24 h or up to 2 days after sample receipt, depending on fixation time. Among cases that could not be resolved by histology or immunohistochemistry alone, Hetairos yielded 63% high-confidence predictions and 37% low-confidence predictions. Hetairos’s top-1 predictions were found to agree with the eventual integrated diagnosis in 120 of 133 high-confidence cases (90.2%; Fig. 7a), highlighting Hetairos’s ability to deliver near-methylation accuracy within a substantially shorter timeframe. For cases with high methylation scores and high confidence, Hetairos achieved an accuracy of 94.3% (100 of 106), while for those with low Hetairos confidence, the accuracy was 45.5% (35 of 77). In this subgroup with low methylation scores, Hetairos and the methylation classifier showed comparable accuracy against the integrated diagnosis (Fig. 7b). Notably, among the low-methylation-score cases that were concordant with Hetairos’s high-confidence predictions, the accuracy reached 88.9% (16 of 18; Fig. 7c), further demonstrating how Hetairos can aid diagnostic decision-making when molecular results are inconclusive.

### Granular tumor classification stratifies survival

Multiple studies have underscored the need for a granular classification of CNS tumors to reflect considerable differences in survival^2,34–37^. To demonstrate the prognostic utility of Hetairos’s detailed classification, we used data from 353 patients with CNS tumors in the MNP 2.0 trial^2^, for whom digital H&E images, methylation classification and survival data are available.

**Fig. 8:**
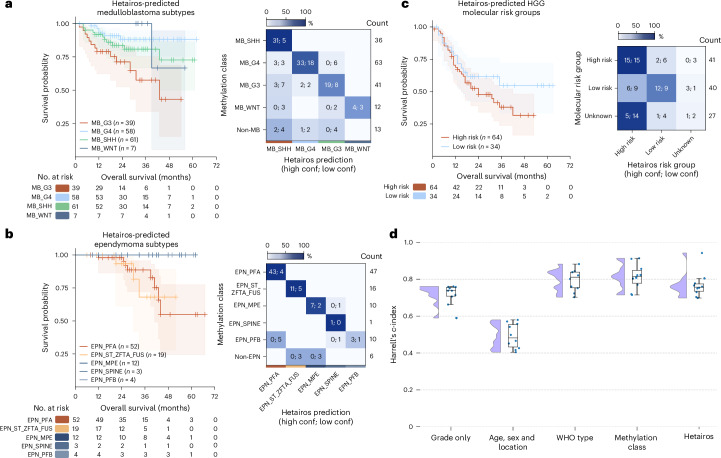
Risk stratification based on predictions by Hetairos. **a**, Analysis of medulloblastoma (*n* = 165 slides). Left, Kaplan–Meier analysis of the subgroups. MB_WNT, medulloblastoma, WNT activated; MB_SHH, medulloblastoma, SHH activated; MB_G3, medulloblastoma, group 3; MB_G4, medulloblastoma, group 4; non-MB, other CNS tumor types. Right, confusion matrix between Hetairos’s predictions and methylation classes. **b**, Ependymoma subtypes (*n* = 90 slides). Left, Kaplan–Meier analysis. EPN_PFA, posterior fossa ependymoma, group PFA; EPN_ST_ZFTA_FUS, supratentorial ependymoma, ZFTA fusion positive; EPN_MPE, myxopapillary ependymoma; EPN_SPINE, spinal ependymoma; EPN_PFB, posterior fossa ependymoma, group PFB; non-EPN, other CNS tumor types. Right, confusion matrix as in **a**. **c**, WHO-defined high-grade gliomas (HGGs) (*n* = 98 slides). Left, Kaplan–Meier curves. Right, confusion matrix for Hetairos’s predictions and methylation classes. In **a**–**c**, shaded areas in Kaplan–Meier plots represent 95% confidence intervals. For the confusion matrices, high- and low-confidence predictions are indicated as pairs (*n*_high_; *n*_low_) in each tile. **d**, Harrell’s *c*-index for different predictor settings. Box plots show the median (center line), 25th–75th percentiles (box bounds) and 1.5× the interquartile range (whiskers). Each dot in the box plot represents the *c*-index for one of the ten outer cross-validation folds. Source data

Among these data, Hetairos classified 165 cases into one of the four WHO-defined subtypes of medulloblastoma: WNT activated, sonic hedgehog (SHH) activated, group 3 and group 4 (Fig. 8a). Compared to the methylation class, the accuracies were 89% and 51% for high- and low-confidence predictions, respectively. Despite some misclassification among low-confidence predictions, Cox proportional hazards models confirmed that Hetairos’s subtypes exhibited notable differences in overall survival (*P* = 0.03). The 3-year overall survival rates were 58% for tumors classified as group 3 medulloblastoma, 81% for SHH-activated medulloblastoma, 88% for group 4 medulloblastoma and 100% for WNT-activated medulloblastoma. These results are in agreement with previous findings^38^.

Similarly, Hetairos predicted 90 cases as five different WHO subtypes of ependymoma (Fig. 8b). The accuracy of high-confidence and low-confidence predictions was 100% and 48%, respectively. Overall survival among the predicted subtypes differed in the expected manner (*P* = 0.07). Group PFA posterior fossa ependymoma and ZFTA fusion-positive supratentorial ependymoma had poorer prognoses, with 3-year survival rates of 89% and 68%, respectively, compared to the other three ependymoma subtypes, all of which had 3-year survival rates of 100%.

Lastly, we applied Hetairos to classify samples histologically diagnosed as high-grade gliomas into more detailed subtypes. These subtypes were grouped into high- and low-risk categories based on independent prior knowledge^2^ (Methods). Notwithstanding the challenges of accurately classifying glioma subtypes, the survival curves exhibited the expected trend of worse overall survival in high-risk groups (3-year survival: 38% versus 55%; *P* = 0.2; Fig. 8c). Additionally, a multivariate survival analysis confirmed that Hetairos provides stronger prognostic value than the clinical baseline alone, offering a more effective alternative in the absence of molecular testing (Fig. 8d).

## Discussion

Here, we present Hetairos, an AI-based algorithm capable of diagnosing 102 types of CNS tumors from scanned H&E slides. Hetairos was trained and evaluated on more than 11,000 slides from 11 institutions across four continents. Crucial to Hetairos’s applicability across cohorts are its realistic confidence estimates, which help judge its prediction accuracy. Depending on the cohort, Hetairos made high-confidence predictions in 50–70% of cases, and those predictions were found to be the correct diagnosis in nearly 90% of instances.

**Extended Data Fig. 10:**
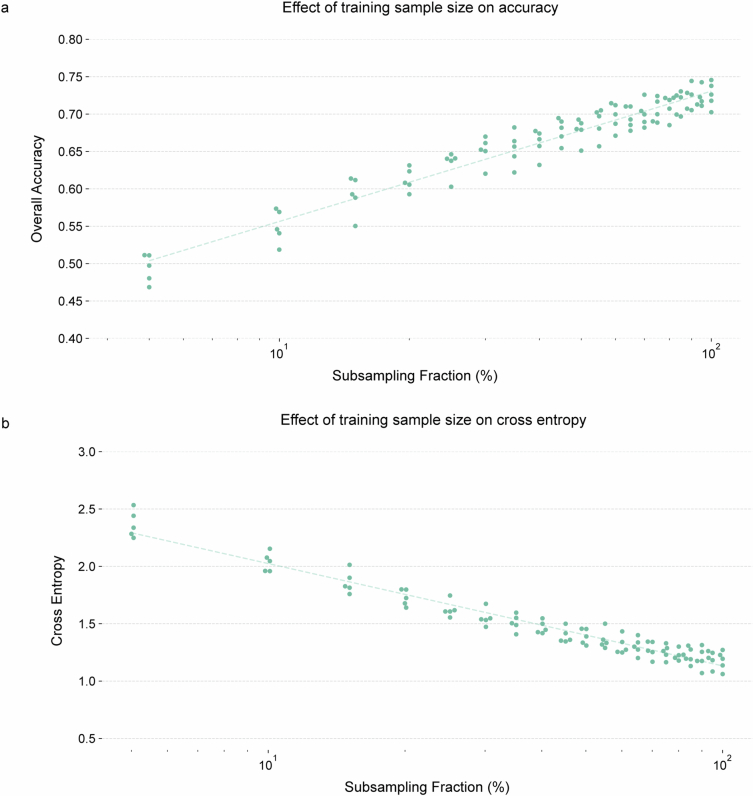
Subsampling experiments. **a**, Scatter plot showing the correlation between training sample size and Hetairos’s predicted overall accuracy. **b**, Scatter plot showing the correlation between training sample size and cross entropy values. Source data

Current limitations of Hetairos include lower confidence and accuracy on rare tumor subtypes, as well as reduced confidence on new cohorts. Both limitations can be mitigated with larger and more diverse training cohorts, as demonstrated by our subsampling experiments (Extended Data Fig. 10a,b and Supplementary Table 12), in which the model’s accuracy increased logarithmically with training size. Hetairos’s performance on external cohorts could be further improved by retraining on data from all cohorts. Further methodological refinements that simulate the effects of different scanners and slide preparation are also likely to improve the algorithm’s performance on new datasets. Moreover, fine-tuning foundation models used for feature extraction—either with cohort-specific data or through parameter-efficient strategies such as LoRA^39^—may further enhance task-specific performance. Currently, Hetairos works with H&E-stained sections of FFPE tissues, as these constitute the diagnostic standard. However, it is conceivable to train extensions that would also work with fresh-frozen tissues, as we observed a decline in model performance on frozen sections.

The utility of Hetairos seems manifold. The ability to provide precise diagnoses with clinically relevant granularity using routine diagnostic material has the potential to enable widespread application. The 2021 WHO classification of CNS tumors requires additional molecular diagnostic tools to cover the full spectrum. This dependence on advanced techniques may restrict comprehensive evaluations to well-resourced healthcare systems^8^. The cost of methylation analysis varies but is approximately €400 per case. While Hetairos uses digital images that require a microscopy slide scanner, such devices are more accessible than those used for methylation profiling. Considering computing resources, energy consumption and other maintenance costs, the estimated average cost per case for running Hetairos is approximately €1–2. Moreover, future Hetairos extensions may enable the use of photographs taken with smartphone adapters for basic microscopes.

Even well-equipped laboratories can benefit from Hetairos, as it helps reduce the time to diagnosis and enables the faster initiation of treatment. We envision Hetairos as a scalable tool for effective diagnostic triaging that may help reduce the number of required molecular tests, thereby lowering costs and freeing up resources. For rare tumor types exhibiting lower accuracies, Hetairos tends to assign low confidence scores, prompting the prioritization of these cases for molecular testing, which is indispensable for their diagnosis. Hetairos can also help establish diagnoses in cases where molecular tests are inconclusive or when there is insufficient material for methylation analysis. The ability to annotate scanned slide images and overlay areas suggestive of a limited set of differential diagnoses guides histopathological assessment and may help train histopathologists to recognize the subtle features that distinguish similar subtypes.

In summary, Hetairos demonstrates the remarkable potential of digital pathology to provide swift and accessible methods for diagnosing tumors with a level of granularity that would otherwise require considerable resources. While Hetairos has—and is aware of—its limitations, these are likely to be overcome in future versions trained on larger and more diverse cohorts and with methodological refinements.

## Methods

### Ethics and inclusion statement

Ethics approval for this study was granted by the Ethics Committee of the Medical Faculty of the University of Heidelberg (S-649/2021). Slides from University College London were obtained under BRAIN UK ethics approval (22/015). Informed consent was waived for this study, and no compensation was provided to participants. Participant selection was determined solely by the availability of histopathology images and corresponding diagnoses. No exclusions were made based on race, ethnicity, sex, gender or other social factors.

### Patient cohorts

Hetairos was developed using 6,115 scanned whole-slide images of H&E-stained CNS tumors from 4,961 cases at UKHD. Slides were scanned using a Leica Aperio AT2 slide scanner at a maximum magnification of 40×. The dataset was divided into 4,803 slides for training, 1,102 slides for internal validation and 210 slides reserved for comparative testing with neuropathologists, with stratification by tumor subtype. There was no overlap of cases between the training and validation sets. Methylation profiles for each tumor were available and used to generate predictions with version 12.8 of the Molecular Neuropathology methylation classifier (https://www.molecularneuropathology.org), which is fully compatible with the Illumina EPIC v1 and v2 (850K) platforms, as well as other sequencing-based platforms, ensuring its continued applicability. A total of 184 methylation-based subclasses were represented in the dataset. The internal validation and comparative testing sets included only samples with methylation scores >0.8. The training set comprised samples with methylation scores ranging from 0.3 to 1. Samples with low methylation scores (<0.8) were included in the training set if the predicted methylation class was consistent with auxiliary molecular findings, such as mutations, fusions or copy number alterations. Methylation classes were aggregated into 102 subtypes to preserve clinically relevant categories while merging those with no established clinical relevance, such as the various subtypes of glioblastoma, which have been shown to display intratumoral variance^40,41^.

In addition to the 20% of the UKHD cohort used as the internal validation set, ten external validation cohorts were analyzed. Methylation classes were used as the ground truth for cohorts with matched methylation classification data. In line with the recommended cutoffs, cases with methylation class scores (MC scores) >0.8 were used and reported in the main text. Cohorts with matched methylation classes were as follows: University College London, UK (*n* = 857; MC score > 0.8: *n* = 535); University Cancer Center Frankfurt, Germany (*n* = 73; MC score > 0.8: *n* = 63); Groupe Hospitalier Universitaire Paris Psychiatrie & Neurosciences, France (*n* = 164; MC score > 0.8: *n* = 134); Justus Liebig University Giessen, Germany (*n* = 217; MC score > 0.8: *n* = 125); Charité Berlin, Germany (*n* = 77; MC score > 0.8: *n* = 77); and The Cancer Genome Atlas (TCGA; *n* = 862; MC score > 0.8: *n* = 732). Cohorts without matched methylation classes were Chongqing Medical University, China (*n* = 70); AC Camargo Cancer Center, Brazil (*n* = 30); Pontificia Universidad Católica de Chile, Chile (*n* = 30; MC score > 0.8: *n* = 30); and the publicly available EBRAINS DBTA (*n* = 3,110). Final integrated diagnoses were used to infer labels for the external validation datasets from Chongqing Medical University and AC Camargo Cancer Center. Histopathological diagnoses provided in the EBRAINS DBTA dataset were matched to the model’s classification scheme where applicable. The DBTA dataset was accessed through the EBRAINS DBTA data portal (https://ebrains.eu, 10.25493/WQ48-ZGX)^42^. Whole-slide images and matched methylation profiles for the TCGA-LGG (low-grade glioma) and TCGA-GBM (glioblastoma) cohorts were downloaded in October 2020 from the Genomic Data Commons data portal^43,44^. Stereotactic biopsy samples and samples from the MNP 2.0 cohort without sufficient material for methylation array analysis or with low methylation scores were included if compatible integrated diagnoses could be issued using other molecular markers, such as characteristic mutations or fusions^2,9^. A total of 353 CNS tumor samples with corresponding H&E images and survival data from the MNP 2.0 cohort were included in the survival difference analysis. These samples primarily consisted of medulloblastomas, ependymomas and high-grade gliomas.

### Hetairos framework

The Hetairos framework consists of three main steps: (1) slide image preprocessing, (2) feature extraction, and (3) feature aggregation and final prediction. Each step is outlined in detail below.

In the preprocessing step, we began by identifying tissue in the slide images. Each whole-slide image was initially segmented to identify tissue regions using hysteresis thresholding at a downsampled resolution. Additionally, we masked ink-covered regions using a manually defined set of colors. Tissue-containing areas were then divided into nonoverlapping tiles of 256 × 256 pixels at a nominal magnification of 20×, corresponding to 0.5 μm per pixel. Finally, we used Sobel image gradient-based filtering to exclude blurred and low-tissue-content tiles.

Subsequently, 1,536-dimensional tile feature representations were extracted using the pretrained Prov-GigaPath model, which is based on a ViT-G/14 vision transformer^45^ pretrained with the DINOv2 framework^46^. The previously obtained tiles were resized to 224 × 224 pixels, resulting in 17.5×-magnification tiles that were then processed using Prov-GigaPath. Notably, other histopathology foundation models could be used as alternatives; in our experiments, UNI performed similarly. After feature extraction, each slide image is represented as an *n* × 1,536 matrix, where *n* represents the number of tiles in the slide.

The feature aggregation step condenses all tile-level features into a single slide-level representation and makes the final prediction by dynamically selecting the most informative tiles. We used a custom-modified TransMIL model, an embedding-based multiple-instance learning (MIL) method for aggregation. The TransMIL architecture consists of two consecutive transformer blocks with Nyström attention^47^, replacing the standard scaled dot-product attention to reduce computational complexity. As a regularization method, we randomly divided the slide matrix $H\in {{\mathbb{R}}}^{n\times 1,536}$ into *m* submatrices, ${H}_{i}\in {{\mathbb{R}}}^{{n}_{i}\times 1,536}({\sum }_{i=1}^{m}{n}_{i}=n)$, which were then separately processed by the aggregator. After applying the multihead Nyström attention (MSA) twice, the resulting embedding matrix can be represented as ${H}_{i}^{\mathrm{attn}}=\mathrm{MSA}(\mathrm{MSA}({H}_{i}))=[{H}_{i}^{\mathrm{cls}};{H}_{i}^{1};\ldots ;{H}_{i}^{{n}_{i}}]$, where ${h}_{i}^{\mathrm{cls}}\in {{\mathbb{R}}}^{1\times 512}$ is the computed class token for submatrix *H*_*i*_, and ${H}_{i}^{j}$ represents the weighted tile embeddings. The slide embedding was subsequently constructed by concatenating the class token from each submatrix, denoted as ${h}_{\mathrm{slide}}=[{h}_{1}^{\mathrm{cls}},\ldots ,{h}_{m}^{\mathrm{cls}}]$, where ${h}_{\mathrm{slide}}\in {{\mathbb{R}}}^{1\times (m\times 512)}$. Each submatrix class token ${h}_{i}^{\mathrm{cls}}$ was processed through a multilayer perceptron (MLP) tailored for localized predictions, while the slide embedding *h*_slide_ was fed into a separate MLP constructed for slide-level predictions. Both outputs were supervised using a unified label, leveraging cross-entropy loss to enforce alignment between local and global predictions and to enhance predictive consistency across scales. Optionally, the patient’s age and the anatomical location of the tumor (extracranial, infratentorial, intra- or periventricular, intra- or suprasellar, pineal, spinal, or supratentorial) can be specified to enhance prediction accuracy. The age is encoded as a 32-dimensional vector using sinusoidal positional encoding functions, while the location is one-hot encoded. These two additional features are concatenated with the class token of each submatrix before being processed by the output MLP.

To further accentuate the differences between the features of various tumor types, feature templates for each tumor class were constructed in the latent space to regulate the interclass feature distances. Specifically, the mean reweighted tile embeddings from each submatrix serve as representation features in the latent space, which is ${z}_{i}=\frac{1}{{n}_{i}}{\sum }_{j=1}^{{n}_{i}}{h}_{i}^{j}$. The corresponding tumor class template feature *Z*_*k*_ is represented as the exponential moving average of the representation features for tumor samples with class *k* given ${Z}_{k}^{l+1}=(1-\alpha ){Z}_{k}^{l}+\alpha {z}_{i}$, where sample *i* belongs to class *k*. Thus, a contrastive loss is formulated based on the class template and representation features in the latent space, as follows:

$${L}_{\mathrm{cl}}=\mathop{\sum }\limits_{i=1,i\in \mathrm{class}\,k}^{n}\left[\left(1-\frac{{z}_{i}}{\mathrm{||}{z}_{i}\mathrm{||}}\frac{{Z}_{k}}{\mathrm{||}{Z}_{k}\mathrm{||}}\right)+\mathop{\sum }\limits_{l\ne k}\min \left(\frac{{z}_{i}}{\mathrm{||}{z}_{i}\mathrm{||}}\frac{{Z}_{l}}{\mathrm{||}{Z}_{l}\mathrm{||}},\varepsilon \right)\right]$$

Combined with the cross-entropy (CE) loss for classification, the overall loss function is defined as follows:

$$L=\mathrm{CE}(\,\hat{y},y)+\frac{1}{m}\mathop{\sum }\limits_{i=1}^{m}\mathrm{CE}(\,{\hat{y}}_{i},y)+\beta {L}_{\mathrm{cl}}$$

where $\hat{y}$ denotes the slide-level prediction, ${\hat{y}}_{i}$ is the local prediction derived from submatrix *H*_*i*_, and *y* corresponds to the class probability vector estimated by version 12.8 of the Molecular Neuropathology methylation classifier.

Data augmentations are widely used to prevent overfitting on training samples. However, directly applying traditional image-level augmentations to tiles in an online manner is computationally expensive within the MIL framework, as the tiles must be passed through the feature extractor each time. To avoid this, we applied augmentation to the training samples in the tile embedding space. We used Mixup^48^ on the feature embeddings generated by the pretrained feature extractor. That is, given the slide embedding matrix *H* with label *k*, and matrix *H*′ with label *k*′ from another sample, the augmented sample is computed as *γH* + (1 − *γH*′), with the corresponding label *γk* + (1 − *γk*′). Note that the tile numbers of the two samples are aligned by downsampling or upsampling before mixing. This augmentation method enhances the diversity of training samples with minimal computational cost.

For inference, we used a deep ensemble to improve predictive accuracy and model robustness by combining the outputs of multiple independently trained models. In this case, ten copies of the aggregator were trained using different seeds. The probability vectors output by the ten models were then averaged to obtain the final predicted probabilities for each tumor subtype. The use of deep ensembles is known to enhance performance, especially for infrequent classes^49^, and to be beneficial for improving the calibration of output probabilities^50^.

The automatically generated reports include heatmaps showing which regions of the slide are predicted to be a given tumor type. This is done by processing small local regions of 5 × 5 tiles in a sliding window fashion and then composing the local predictions into the global prediction map.

### Implementation details

The Hetairos model was implemented with Python 3.10.15 using the PyTorch Lightning (version 2.0.8) and PyTorch (version 2.5.1) frameworks. The libraries mainly involved were OpenSlide (version 1.2.0), OpenCV (version 4.8.0), Timm (version 1.0.8), Pillow (version 9.5.0), NumPy (version 1.24.4), pandas (version 2.0.3), h5py (version 3.9.0) and Wandb (version 0.18.7). During training, the Hetairos aggregator weights were initialized with a random seed, and a batch size of 1 was used. We used a Lookahead optimizer with a learning rate of 1 × 10^−5^ and a weight decay of 1 × 10^−5^ to optimize the model weights, with an early stopping threshold set at 10 epochs. The number of random submatrices *m* was set to 3. The margin parameter *ε* in the contrastive loss was set to 0.2, and its corresponding weight coefficient *β* was set to 20. The smoothing factor *α* used for calculating the exponential moving average was dynamically adjusted to 0.5, 0.75 and 0.99 during epochs 0–3, 6–9 and 9 onward, respectively. The data augmentation probability was set to 0.7, while age and anatomical location data were randomly omitted at a rate of 0.3. The training and testing of Hetairos can be performed on consumer-grade hardware, such as a single GPU with at least 11 GB of graphics memory (for example, a GeForce RTX 2080 Ti). There are no stringent requirements for the CPU; however, a higher thread count may enhance the efficiency of tiling during the preprocessing step. Additionally, a system with more than 16 GB of RAM is recommended.

### Neuropathologists versus Hetairos

A total of 210 whole-slide images were sent on portable hard drives to five board-certified neuropathologists. Each class represented in the dataset comprised one to three slides. The neuropathologists were asked to select up to three diagnoses from a drop-down list based on their level of confidence in each diagnosis. No additional information about the cases, beyond the H&E slide images, was provided to the neuropathologists or the model.

### Risk groups for survival stratification

For the Hetairos risk groups, model-predicted classes were used to establish the high- and low-risk groups for high-grade gliomas based on prior knowledge^2^. The high-risk group consisted of the following tumors: diffuse midline glioma, H3 K27 altered; diffuse hemispheric glioma, H3 G34 mutant; high-grade diffuse glioma of the midline/posterior fossa, H3/IDH wild-type; diffuse pediatric-type high-grade glioma; and glioblastoma, IDH wild-type. The low-risk group included the following tumors: astrocytoma, IDH mutant; pleomorphic xanthoastrocytoma; infant-type hemispheric glioma; CNS neuroblastoma, FOXR2 altered; HGAP; pilocytic astrocytoma; ganglioglioma; diffuse glioma, MYB or MYBL1 altered; and diffuse leptomeningeal glioneuronal tumor. Predictions falling outside the defined categories were classified as ‘unknown’. For the molecular risk group defined by Sturm et al.^2^, the intermediate-risk group was merged into the low-risk group to facilitate analysis.

### Statistics and reproducibility

No statistical method was used to predetermine the sample size; all available cases from participating centers that met the inclusion criteria were included. No data were excluded from the analysis. The internal dataset was split into training (80%) and validation (20%) sets using random patient-level assignment. External validation cohorts were not randomized. Data collection and analysis were not performed blind to the conditions of the experiments. Data distribution was not assumed to be normal for the main classification performance analyses, which were based on categorical outcomes and nonparametric summary metrics, including top-1 accuracy, top-3 accuracy and confidence-stratified accuracy. Differences in overall survival across Hetairos-predicted subtypes were assessed using a Cox proportional hazards model, with statistical significance determined by the likelihood ratio test, which does not assume a specific distribution of the survival data. The relevant model assumptions are proportional hazards, independent observations and noninformative censoring. Hetairos is a deterministic model with fixed weights; given identical inputs, its predictions are fully reproducible.

### Reporting summary

Further information on research design is available in the Nature Portfolio Reporting Summary linked to this article.

## Supplementary information

Reporting Summary

Peer Review File

Supplementary Tables Supplementary Tables 1–12.

## Source data

Source Data Fig. 1 Statistical source data.

Source Data Fig. 2 Statistical source data.

Source Data Fig. 3 Statistical source data.

Source Data Fig. 4 Statistical source data.

Source Data Fig. 5 Statistical source data.

Source Data Fig. 6 Statistical source data.

Source Data Fig. 8 Statistical source data.

Source Data Extended Data Fig. 1 Statistical source data.

Source Data Extended Data Fig. 2 Statistical source data.

Source Data Extended Data Fig. 3 Statistical source data.

Source Data Extended Data Fig. 4 Statistical source data.

Source Data Extended Data Fig. 5 Statistical source data.

Source Data Extended Data Fig. 8 Statistical source data.

Source Data Extended Data Fig. 9 Statistical source data.

Source Data Extended Data Fig. 10 Statistical source data.

## Data Availability

Restrictions apply to the availability of datasets used for training and internal validation, which were collected retrospectively under institutional and ethical approval and are, therefore, not publicly available. These datasets comprise routine clinical histopathology and DNA methylation data collected as part of the standard diagnostic workup and were not generated for research purposes; therefore, they are not eligible for public redistribution. Requests for these datasets should be directed to one of the corresponding authors (F.S., felix.sahm@med.uni-heidelberg.de). Requests will be reviewed within 4 weeks and evaluated according to institutional and departmental policies regarding patient privacy and intellectual property obligations. External validation datasets were obtained under data sharing agreements with the respective contributing institutes and cannot be redistributed. Access requests should be directed to the respective contributing institutions. Publicly available datasets used in this study included TCGA-LGG, TCGA-GBM and the Digital Brain Tumour Atlas (EBRAINS DBTA). Additional data supporting the findings and reproducibility of this study are available at https://github.com/gerstung-lab/Hetairos. Source data are provided with this paper.

## Code availability

The code for Hetairos training and validation, data preprocessing and figure reproduction is publicly available at https://github.com/gerstung-lab/Hetairos.
