# Supplementary material for: Hetairos is a histology-based artificial intelligence model for predicting central nervous system tumor methylation subtypes
Source: Nat Cancer. 2026 Jun 10;7(6):884–98. doi: 10.1038/s43018-026-01186-3 (PMC13309283; doi:10.1038/s43018-026-01186-3)
Supplement: Supplementary file 1 — Reporting Summary [file 43018_2026_1186_MOESM1_ESM.pdf]

nature portfolio

Corresponding author(s): Felix Sahm and Moritz Gerstung

Last updated by author(s): Mar 9, 2026

# Reporting Summary

Nature Portfolio wishes to improve the reproducibility of the work that we publish. This form provides structure for consistency and transparency in reporting. For further information on Nature Portfolio policies, see our Editorial Policies and the Editorial Policy Checklist.

## Statistics

For all statistical analyses, confirm that the following items are present in the figure legend, table legend, main text, or Methods section.

| n/a | Confirmed | |
|---|---|---|
| ☐ | ☒ | The exact sample size (*n*) for each experimental group/condition, given as a discrete number and unit of measurement |
| ☒ | ☐ | A statement on whether measurements were taken from distinct samples or whether the same sample was measured repeatedly |
| ☒ | ☐ | The statistical test(s) used AND whether they are one- or two-sided<br>*Only common tests should be described solely by name; describe more complex techniques in the Methods section.* |
| ☒ | ☐ | A description of all covariates tested |
| ☒ | ☐ | A description of any assumptions or corrections, such as tests of normality and adjustment for multiple comparisons |
| ☐ | ☒ | A full description of the statistical parameters including central tendency (e.g. means) or other basic estimates (e.g. regression coefficient) AND variation (e.g. standard deviation) or associated estimates of uncertainty (e.g. confidence intervals) |
| ☒ | ☐ | For null hypothesis testing, the test statistic (e.g. *F*, *t*, *r*) with confidence intervals, effect sizes, degrees of freedom and *P* value noted<br>*Give P values as exact values whenever suitable.* |
| ☒ | ☐ | For Bayesian analysis, information on the choice of priors and Markov chain Monte Carlo settings |
| ☒ | ☐ | For hierarchical and complex designs, identification of the appropriate level for tests and full reporting of outcomes |
| ☒ | ☐ | Estimates of effect sizes (e.g. Cohen's *d*, Pearson's *r*), indicating how they were calculated |

*Our web collection on statistics for biologists contains articles on many of the points above.*

## Software and code

Policy information about availability of computer code

| | |
|---|---|
| Data collection | No software/code was used to collect data. |
| Data analysis | Algorithms of this study were mostly programmed with Python (version 3.10.15) and libraries mainly involved were mainly OpenSlide (version 1.2.0), OpenCV (version 4.8.0), Timm (version 1.0.8), Pillow (9.5.0), NumPy (version 1.24.4), pandas (version 2.0.3), h5py (version 3.9.0) and Wandb (0.18.7).<br>The preprocessing, feature extraction and training/test code of Hetairos can be accessed at https://github.com/gerstung-lab/Hetairos, along with the Jupyternotebook to reproduce the figures in the manuscript. |

For manuscripts utilizing custom algorithms or software that are central to the research but not yet described in published literature, software must be made available to editors and reviewers. We strongly encourage code deposition in a community repository (e.g. GitHub). See the Nature Portfolio guidelines for submitting code & software for further information.

## Data

Policy information about availability of data

All manuscripts must include a data availability statement. This statement should provide the following information, where applicable:

- Accession codes, unique identifiers, or web links for publicly available datasets
- A description of any restrictions on data availability
- For clinical datasets or third party data, please ensure that the statement adheres to our policy

Restrictions apply to the availability of datasets used for training and internal validation, which were collected retrospectively under institutional and ethical approval and are therefore not publicly available. These datasets comprise routine clinical histopathology and DNA methylation data collected as part of standard diagnostic workup, and were not generated for research purposes; they are therefore not eligible for public redistribution. Requests for these datasets should be directed to the corresponding author at felix.sahm@med.uni-heidelberg.de. Requests will be reviewed within 4 weeks and evaluated according to institutional and departmental policies regarding patient privacy and intellectual property obligations. External validation datasets were obtained under data sharing agreements with the respective contributing institutes and cannot be redistributed. Access requests should be directed to the respective contributing institutes. Publicly available datasets used in this study included TCGA-LGG, TCGA-GBM and The Digital Brain Tumour Atlas (EBRAINS DBTA). Source data for Figures 1–8 and Extended Data Figures 1–10 have been provided as Source Data files. Additional data supporting the findings and reproducibility of this study are available at https://github.com/gerstung-lab/Hetairos.

## Research involving human participants, their data, or biological material

Policy information about studies with human participants or human data. See also policy information about sex, gender (identity/presentation), and sexual orientation and race, ethnicity and racism.

| | |
|---|---|
| Reporting on sex and gender | Of the 5,905 samples used for training and internal validation, sex information was available for 4,967 samples (male:female=2,674:2,293), with 2,169:1,854 in the training set and 505:439 in the validation set. Of the 210 samples included in the Hetairos versus neuropathologist comparison, sex information was available for 176 samples (male: female = 97:79). Of the 857 samples included in UCL dataset, sex information was available for 543 samples (male:female=280:263) with 226:200 in the high-methylation-score subset and 63:54 in the low-methylation-score subset. For all other cohorts, samples were anonymised and corresponding sex information was not available. No exclusions were made based on race, ethnicity, sex, gender, or other social factors. |
| Reporting on race, ethnicity, or other socially relevant groupings | Participant selection was solely determined by the availability of histopathology images and matched diagnoses. No exclusions were made based on race, ethnicity, sex, gender, or other social factors. We did not independently collect, modify, or interpret these demographic variables. |
| Population characteristics | Participant selection was solely determined by the availability of histopathology images and matched diagnoses. No exclusions were made based on race, ethnicity, sex, gender, or other social factors. |
| Recruitment | Informed consent is waived for this study and no participant compensation was provided. |
| Ethics oversight | Ethics Committee of the Medical Faculty of the University of Heidelberg (S-649/2021). Slides from University College London were obtained under the BRAIN UK ethics approval (22/015). |

Note that full information on the approval of the study protocol must also be provided in the manuscript.

# Field-specific reporting

Please select the one below that is the best fit for your research. If you are not sure, read the appropriate sections before making your selection.

☒ Life sciences ☐ Behavioural & social sciences ☐ Ecological, evolutionary & environmental sciences

For a reference copy of the document with all sections, see nature.com/documents/nr-reporting-summary-flat.pdf

# Life sciences study design

All studies must disclose on these points even when the disclosure is negative.

| | |
|---|---|
| Sample size | The study included 6115 slides (4961 tumours) from the UKHD dataset and 5498 slides (4645 tumours) from the external dataset. The number of datasets was determined by the availability of digitised H&E slides with matched methylation classifications from institutions. |
| Data exclusions | No data exclusions were performed. |
| Replication | The codes for replication including preprocessing and training/testing of Hetairos, are available at https://github.com/gerstung-lab/Hetairos. |
| Randomization | Samples were randomly assigned to 80% of the training and 20% of the validation for the UKHD dataset. |
| Blinding | Blinding was not applicable as this study mainly involved computational analysis of retrospective data without any experimental intervention |

Blinding | or outcome assessment by human observers. For the prospective validation component, AI-based predictions were generated independently of and without access to clinical diagnostic workflows.

# Reporting for specific materials, systems and methods

We require information from authors about some types of materials, experimental systems and methods used in many studies. Here, indicate whether each material, system or method listed is relevant to your study. If you are not sure if a list item applies to your research, read the appropriate section before selecting a response.

## Materials & experimental systems

| n/a | Involved in the study |
| --- | --- |
| ☒ | ☐ Antibodies |
| ☒ | ☐ Eukaryotic cell lines |
| ☒ | ☐ Palaeontology and archaeology |
| ☒ | ☐ Animals and other organisms |
| ☒ | ☐ Clinical data |
| ☒ | ☐ Dual use research of concern |
| ☒ | ☐ Plants |

## Methods

| n/a | Involved in the study |
| --- | --- |
| ☒ | ☐ ChIP-seq |
| ☒ | ☐ Flow cytometry |
| ☒ | ☐ MRI-based neuroimaging |

## Plants

Seed stocks

*Report on the source of all seed stocks or other plant material used. If applicable, state the seed stock centre and catalogue number. If plant specimens were collected from the field, describe the collection location, date and sampling procedures.*

Novel plant genotypes

*Describe the methods by which all novel plant genotypes were produced. This includes those generated by transgenic approaches, gene editing, chemical/radiation-based mutagenesis and hybridization. For transgenic lines, describe the transformation method, the number of independent lines analyzed and the generation upon which experiments were performed. For gene-edited lines, describe the editor used, the endogenous sequence targeted for editing, the targeting guide RNA sequence (if applicable) and how the editor was applied.*

Authentication

*Describe any authentication procedures for each seed stock used or novel genotype generated. Describe any experiments used to assess the effect of a mutation and, where applicable, how potential secondary effects (e.g. second site T-DNA insertions, mosiacism, off-target gene editing) were examined.*
