# Supplementary material for: Hetairos is a histology-based artificial intelligence model for predicting central nervous system tumor methylation subtypes
Source: Nat Cancer. 2026 Jun 10;7(6):884–98. doi: 10.1038/s43018-026-01186-3 (PMC13309283; doi:10.1038/s43018-026-01186-3)
Supplement: Supplementary file 2 — Peer Review File [file 43018_2026_1186_MOESM2_ESM.pdf]

nature portfolio

Peer Review File

Hetairos is a histology-based artificial intelligence model for predicting central nervous system tumour methylation subtypes

Corresponding Author: Professor Moritz Gerstung

This manuscript has been previously reviewed at another journal. This document only contains information relating to versions considered at Nature Cancer.

This file contains all reviewer reports in order by version, followed by all author rebuttals in order by version.

Version 0:

Reviewer comments:

Reviewer #2

(Remarks to the Author)
Revisions are acceptable.

(Remarks on code availability)
Not qualified to evaluate code.

Reviewer #3

(Remarks to the Author)
The authors of the Hetairos manuscript have submitted a revised version in which they provided detailed responses to each of the criticisms from the original review. However, the responses leave open most of the identified weaknesses and mitigate only minimally the previously expressed concerns. Some examples of substantially unaddressed points are:
- The AI model continues to have rather low accuracy for rare classes of tumors. The low accuracy scores have not been improved by either oversampling and color space augmentation. Consequently, the broad utility of the model remains uncertain as it seems to perform accurately only for the most frequent classes of tumors that are enriched with high-confidence samples and present limited diagnostic challenges.
- The confidence drop of Hetairos in external cohorts has not been mitigated in the revised manuscript. The authors offer a lengthy discussion trying to explain the reasons behind such drop in confidence but it remains unclear whether those statements have addressed the substance for any of the points raised during the first round of review.
- The mismatches occurring for some cases between the predictions by Hetairos and those resulting from the DNA methylation classifier remain unexplained. Ultimately, it is unclear whether, in those instances, users should trust Hetairos or the DNA methylation classifier.
- While the authors provide a few anecdotal examples of the ability of Hetairos to discriminate between areas with high from low Ki67 or vascular proliferation, whether those features are robustly and consistently predicted by Hetairos across each of the different tumor classes remains uncertain. As presented, the tumor subtypes from which the algorithm is particularly robust seem to select instances of obvious and simplistic distinctions.

(Remarks on code availability)

Reviewer #4

(Remarks to the Author)
The authors present Hetairos, an AI-based framework that classifies 102 central nervous system (CNS) tumor subtypes directly from H&E-stained slides. The model was trained and validated on >11,000 slides from 11 institutions across 4 continents. Hetairos achieves high-confidence predictions in 50-70% of cases with ~0.87 accuracy, outperforming

neuropathologists in direct comparison. A prospective clinical evaluation showed near-methylation level accuracy with a turnaround time of ~12 minutes, compared to ~12 days for molecular testing. The tool also provides interpretable prediction maps and demonstrates prognostic value by stratifying patient survival within medulloblastoma, ependymoma, and glioma subtypes.

The paper is well-written by authors who have a firm grasp of both neuropathology and computational pathology. The work is the largest study to date investigating the role of AI for predicting methylation status in CNS tumors.

The authors have provided a thorough, point-by-point reply to all of Reviewer 1's concerns:
Rare tumor classes: They implemented oversampling and multiple augmentation strategies (color jittering, stain normalization, random stain transformations), but these provided only marginal gains. They acknowledge performance remains poor in classes with <20 examples and emphasize the need for larger datasets.
Domain shift/external validation: They quantified differences in staining/scanner protocols (RGB distributions, PCA of color embeddings, scanner model table) and tested stain normalization as a domain adaptation strategy. This did not improve performance, but they transparently discuss the limitation.
Low-confidence predictions: They now explicitly propose that such cases be flagged for molecular testing, using top-3 predictions to guide targeted assays.
Prospective/real-world workflow: While not yet tested prospectively with pathologists receiving AI output, they expanded the prospective cohort to 210 cases and discuss plans for real-time workflow integration.
Heatmaps: They validated heatmap interpretability with pathologists on 16 cases, finding that low-confidence cases often correspond to misleading heatmaps, but that high-confidence heatmaps were reliable.
Hierarchical classification: They implemented two hierarchical approaches but found no consistent improvement over flat classification, and report these results in detail.
Discrepancies with methylation profiling: They analyzed mismatches, showing Hetairos sometimes agreed with final diagnoses when methylation did not, suggesting potential complementarity.
Pathologist vs. AI on rare tumors: They confirmed Hetairos struggles with classes with <10 cases, where pathologists outperform. They attribute this to sample size and emphasize dataset expansion as the solution.
Intra-tumoral heterogeneity: They performed additional analyses with manual tumor/healthy annotations, demonstrating robustness but variability in confidence at low tumor fractions.
Survival analysis: They conducted a multivariate survival analysis (Cox models with c-index), showing Hetairos adds prognostic value beyond baseline clinical features.
Robustness to artifacts: They evaluated artifacts under strict QC and showed Hetairos maintained performance.
Demographic/center bias: They reported center-specific scanner/staining differences and their impact.
FFPE vs. frozen tissue: This remains speculative, but they note Hetairos was trained on FFPE and could in principle be adapted.
Overall, the rebuttal convincingly demonstrates that the authors addressed each of Reviewer 1's comments. In most cases, they performed additional analyses, updated figures, or clearly acknowledged limitations. I find that the authors have responded adequately and in good faith to all of Reviewer 1's comments, with substantial new analyses, figures, and clarifications.

However, Reviewer 3 is correct that some of the underlying weaknesses (rare class performance, external generalization, AI vs. methylation discordances) remain unresolved at a substantive level.

While reviewing the revised manuscript, I also noted some points that would strengthen the study, as mentioned below:
Major comments
1)For the main analysis, the authors have divided the cases into high and low confidence, which is a good choice, especially given that the authors have demonstrated that the classifier is well calibrated. I recommend that the authors report model performance metrics over the full testing sets that are not susceptible to class imbalance. Mean class accuracy is a good metric and will give the readers a sense of how well the model performs on a tumor class basis (rather than an instance basis). These metrics can be in extended or supplemental data figures.
2) The authors perform a thorough analysis of model performance, especially related to model uncertainty. One interesting additional analysis would be to decompose the model uncertainty into epistemic and aleatoric uncertainty. Morphologic features alone are likely insufficient to predict (at least some) methylation classes at 100% accuracy, which means the aleatoric uncertainty will continue to dominate regardless of the amount of additional training examples. Importantly, the upper bound on histopath-based methylation classification from WSIs is still unknown. The authors are in a good position to shed some light on the source of uncertainty. I leave the decision to the authors if this is feasible.
3) The authors used a pretrained foundation model for feature extraction. Prov-Gigapath is trained using self-supervision. We have found that self-supervised CPath models are often suboptimal for molecular classification tasks and overfit to morphological features. Did the authors consider fine-tuning the feature extractor using parameter efficient fine tuning (Adapters, LORA, etc.)? With ViT-G this may be challenging, but I would consider trying to better optimize the feature extraction for the task.
Minor comments
1) Please include an ablation on the performance without age and tumor location. It is helpful and informative to know how much these factors contribute to the prediction. These results should be in extended data figures.
2) The paper is very methylation-focused, but this does not appear in the title and is slightly misleading. Consider adding this.
Great work. Strong paper that could only be executed by the authors.

(Remarks on code availability)

Version 1:

Reviewer comments:

Reviewer #4

(Remarks to the Author)
The authors are commended for their revision. They did a good job reporting additional metrics. They did due diligence on uncertainty quantification, which is not a straightforward analysis. I especially liked the new scaling experiments. The log-linear relationship between positive examples and performance is expected. They are also correct in pointing out that some classes are unlikely to improve with additional training because they are either too hard or too easy to classify using histologic features. Overall, the manuscript is stronger, and all my questions have been addressed.

(Remarks on code availability)

Open Access This Peer Review File is licensed under a Creative Commons Attribution 4.0 International License, which permits use, sharing, adaptation, distribution and reproduction in any medium or format, as long as you give appropriate credit to the original author(s) and the source, provide a link to the Creative Commons license, and indicate if changes were made.
In cases where reviewers are anonymous, credit should be given to 'Anonymous Referee' and the source.
The images or other third party material in this Peer Review File are included in the article's Creative Commons license, unless indicated otherwise in a credit line to the material. If material is not included in the article's Creative Commons license and your intended use is not permitted by statutory regulation or exceeds the permitted use, you will need to obtain permission directly from the copyright holder.
To view a copy of this license, visit https://creativecommons.org/licenses/by/4.0/

RE: (NATCANCER-A17980-T), "AI-based histopathological classification of central nervous system tumours"

We sincerely appreciate the feedback from the reviewers regarding our work. Based on their comments, we have implemented additional analyses as suggested and add the corresponding results to the revised manuscript and supplementary materials.

The main changes in the revised manuscript include:

- Further discussion of the strengths and weaknesses of Hetairos and its envisaged role as a supplementary diagnostic tool to address Reviewer 3's conceptual concerns.
- We have reported the class-balanced metrics for the internal and external validation requested by Reviewer 4.
- We have conducted the uncertainty analysis requested by Reviewer 4 and explored the relationship between training sample size and accuracy.
- We have added the ablation study results on age and location information requested by Reviewer 4.

In the following, we provide point-by-point replies to all reviewer comments. We use *italic* fonts for the reviewer comments and Arial font for our replies. Changes made to the manuscript are highlighted in red.

Referee #3:

1 - The AI model continues to have rather low accuracy for rare classes of tumors. The low accuracy scores have not been improved by either oversampling and color space augmentation. Consequently, the broad utility of the model remains uncertain as it seems to perform accurately only for the most frequent classes of tumors that are enriched with high-confidence samples and present limited diagnostic challenges.

We appreciate the reviewer's comment and fully recognise that Hetairos' performance on rare tumour types remains a limitation of the current model, mainly due to the scarcity of training samples for rare tumour classes - often fewer than 5 samples per subtype. On the other hand, Hetairos resolves clinically relevant subtypes of medulloblastoma, ependymoma and meningioma that can currently only be reliably detected by molecular testing, thus demonstrating the utility of the tool in challenging diagnostic questions.

As clarified in the revised text, the 12 tumour classes with accuracy below 0.25 were those with fewer than 20 training samples. The limited number of samples in these cases remains an inherent issue as a result of ultra-rare prevalence of these tumour subtypes. We note, however, that Hetairos correctly flags these hard-to-diagnose ultra-rare cases by assigning a low confidence to the class predictions. This is beneficial as those cases can then be prioritised for more extensive molecular testing to establish the correct diagnosis.

We would also like to add that these rare classes only constitute a very small proportion of the clinical cases, while the model is designed to serve as an efficient and scalable tool for initial diagnostic triage. Even though integrated diagnosis remains essential for final classification of rare entities, the proposed approach can substantially reduce time, labour, and resource requirements and help narrow down the possible classes during the initial diagnostic workflow.

It's also demonstrated in **Figure 5** that Hetarios consistently outperforms human neuropathologists showing its capabilities go beyond easy-to-diagnose tasks.

We have made these points clearer in the revised manuscript text on page 29, including the model's intended role, limitations and potential strategies for mitigation:

"Both limitations can be resolved with larger and more diverse training cohorts, as also evidenced by our subsampling experiments (**Extended Data Figure 10** and **Supplementary Table 12**), where model accuracy increases logarithmically with training size."

"For rare tumour types exhibiting lower accuracies, Hetairos has a tendency to assign low confidence scores, prompting prioritisation of these cases for molecular testing, which is indispensable for their diagnosis."

2 - The confidence drop of Hetairos in external cohorts has not been mitigated in the revised manuscript. The authors offer a lengthy discussion trying to explain the reasons behind such drop in confidence but it remains unclear whether those statements have addressed the substance for any of the points raised during the first round of review.

We appreciate the reviewer’s comment and have clarified that the confidence difference mainly reflects domain shift, a widely recognised and yet unresolved challenge in computational pathology (and across the broader machine learning field). As shown in **Extended Data Figure 4**, differences in staining, slide preparation and scanner characteristics are some potential sources of this shift.

It’s also worth noting that the decrease in model confidence also indicates that it remains well-calibrated on external validation, thereby effectively avoiding overconfident mispredictions.

In the revised manuscript, we have strengthened this explanation and discussed future directions on page 29 as follows,

“Both limitations can be resolved with larger and more diverse training cohorts, as also evidenced by our subsampling experiments (**Extended Data Figure 10** and **Supplementary Table 12**), where model accuracy increases logarithmically with training size.”

“Further methodological refinements which simulate the effect of different scanners and slide preparation are also likely to improve the algorithm's performance in new data sets.”

3 - The mismatches occurring for some cases between the predictions by Hetairos and those resulting from the DNA methylation classifier remain unexplained. Ultimately, it is unclear whether, in those instances, users should trust Hetairos or the DNA methylation classifier.

We understand the reviewer's concern and will clarify in the revised version that Hetairos is designed as **a complementary diagnostic assistant** rather than a replacement for methylation analysis. The typical neuropathological diagnostic process does not rely on a single diagnostic approach, but combines results from histopathological assessment, immunohistochemistry, methylation analysis and also genomic sequencing in an integrated diagnosis. We envisage Hetairos to become a further element of this decision-making process rather than a replacement.

In practice, when there is a discrepancy between high-confidence predictions from Hetairos and those from methylation profiling, the final integrated diagnosis is made by the neuropathologist, taking into account results from additional tests. In our prospective study, for cases where the DNA methylation classifier assigned a low-confidence diagnosis, Hetairos' high-confidence predictions were consistent with the final integrated diagnosis (which was made without input from Hetairos) in 89% of instances, also highlighting its potential as a complementary tool.

We have added the description to clarify Hetairos' role as a supplementary tool in the revised text as follows,

p.20: "Hetairos fits into this workflow as a tool to supplement the first-line of manual histopathological evaluation with H&E stained FFPE slides. Owing to its WHO 2021 compatible granularity and well-calibrated predictions, it is intended to be used as a triaging tool to efficiently inform further molecular analyses."

p.29: "We envision Hetairos to be used as a scalable tool for effective diagnostic triaging, which may help reduce the number of required molecular tests and thereby reduce costs and free up resources."

4 - While the authors provide a few anecdotal examples of the ability of Hetairos to discriminate between areas with high from low Ki67 or vascular proliferation, whether those features are robustly and consistently predicted by Hetairos across each of the different tumor classes remains uncertain. As presented, the tumor subtypes from which the algorithm is particularly robust seem to select instances of obvious and simplistic distinctions.

We recognise the reviewer's concern and would like to clarify that some of the examples were selected to remain accessible to a broad readership and may thus appear to be overly simple. We also appreciate that we have provided relatively little discussion about the nature of the observed patterns. The selected examples were chosen to demonstrate that Hetairos captures a broad range of histopathological characteristics in diverse diagnoses. For meningioma there was frequent intra-tumour heterogeneity observed by Hetairos, which we confirmed in a selected case by Ki67 expression. Importantly, the model's ability to accurately classify over a hundred CNS tumour subtypes indicates that the learned representations extend far beyond simple contrasts between tumour and normal tissue, capturing more complex and biologically relevant morphological variations. The head-to-head comparison with neuropathologists further supports this: Hetairos achieves an accuracy of 68% compared with 30% for human experts, underscoring that its performance is not limited to trivial or overtly distinguishable cases.

We appreciate, of course, that the presented examples are heterogeneous – as a consequence of Hetairos' versatility – and may appear somewhat unconnected and anecdotal. We have therefore restructured this section of the text to become more coherent.

Lastly, we are conscious that these observations are not as extensively validated as other sections of the manuscript, which is why we are presenting these results as Extended Data.

In the revised manuscript, we provide further details on the selected examples on page 20.

"Within the spectrum of glial morphology, pilocytic astrocytoma bulk tissue is clearly distinguished from surrounding reactive gliosis (**Figure 6b**). Similarly, Hetairos uniformly identifies the prototypical histology of oligodendroglioma (**Extended Data Figure 6**). In astrocytoma, areas considered by Hetairos to be indicative of the second-best diagnosis, glioblastoma, coincide with microvascular proliferation, which is a typical feature of both diagnoses and criterion for grade 4 (**Extended Data Figure 7**).

Further examples of intra-tumour heterogeneity are found in meningiomas, where Hetairos frequently identifies regions assigned to the benign category within cases of the intermediate group and vice versa (**Extended Data Figure 8**). The biological

underpinning of the distinct grading classes found within the same tumour is supported by patterns of Ki-67 staining, which is lower in areas predicted to be benign. Taken together, these examples illustrate that Hetairos has the potential to capture subtle but clinically relevant and biologically meaningful histologies, which exhibit intra-tumour heterogeneity and pose diagnostic challenges.”

Referee 4:

I find that the authors have responded adequately and in good faith to all of Reviewer 1's comments, with substantial new analyses, figures, and clarifications.

However, Reviewer 3 is correct that some of the underlying weaknesses (rare class performance, external generalization, AI vs. methylation discordances) remain unresolved at a substantive level.

While reviewing the revised manuscript, I also noted some points that would strengthen the study, as mentioned below:
Major comments

1 - For the main analysis, the authors have divided the cases into high and low confidence, which is a good choice, especially given that the authors have demonstrated that the classifier is well calibrated. I recommend that the authors report model performance metrics over the full testing sets that are not susceptible to class imbalance. Mean class accuracy is a good metric and will give the readers a sense of how well the model performs on a tumor class basis (rather than an instance basis). These metrics can be in extended or supplemental data figures.

We thank Reviewer 4 for the constructive comment regarding model performance metrics. Our current manuscript already includes class-wise accuracies visualized through confusion matrices for both internal and external validation cohorts (**Figure 3** and **Extended Data Figure 3**), enabling readers to assess the performance at the individual tumour-class level.

In addition, as suggested, we have complemented these results by reporting additional metrics less affected by class imbalance including mean class accuracy, Cohen's Kappa and Matthews Correlation Coefficient as presented in **Table S5**.

Table S5

CLASSIFICATION METRICS ACROSS DIFFERENT VALIDATION SETS.

	Mean class accuracy	Cohen's Kappa	Matthews Correlation Coefficient
UKHD validation	0.474/0.662	0.730/0.865	0.731/0.866
External validation	0.388/0.559	0.648/0.849	0.651/0.850
Human vs. Hetairos	0.638/0.850	0.672/0.877	0.675/0.878

Note: The numbers in each unit correspond to metrics for all predictions and high-confidence predictions.

We have supplemented the statement about the abovementioned metrics in the revised manuscript:

p.8: “Top-1 predictions are in agreement with methylation classification results (methylation score > 0.8) in 75% of all internal validation tumours (**Figure 2b**) and remain robust in the presence of common histological artifacts (**Supplementary Tables 3, 4**), with additional performance metrics provided in **Supplementary Table 5**.”

p.13: “Hetairos’s overall accuracy was lower in external validation cohorts than in the internal validation cohort (68% and 75% respectively) (**Figure 4c**, **Extended Data Figure 3** and **Supplementary Table 5**).”

p.18: “Based on H&E-stained sections only, Hetairos’s accuracy was uniformly better than that of neuropathologists who achieved an average top-1 accuracy of 0.33 (0.18-0.39) compared to Hetairos’s 0.68 (additional metrics provided in **Supplementary Table 5**).”

2 - The authors perform a thorough analysis of model performance, especially related to model uncertainty. One interesting additional analysis would be to decompose the model uncertainty into epistemic and aleatoric uncertainty. Morphologic features alone are likely insufficient to predict (at least some) methylation classes at 100% accuracy, which means the aleatoric uncertainty will continue to dominate regardless of the amount of additional training examples. Importantly, the upper bound on histopath-based methylation classification from WSIs is still unknown. The authors are in a good position to shed some light on the source of uncertainty. I leave the decision to the authors if this is feasible.

We appreciate the insightful suggestion from the reviewer. Decomposing the total entropy of a prediction into aleatoric and epistemic components is a concept rooted in Bayesian machine learning, where the model parameters are treated as a distribution conditioned on the training data, i.e. $p(y|x, D) = \int p(y|x, \theta)p(\theta|D)\, d\theta$. In practice, exact computation of this posterior is intractable for deep neural networks, so we approximate it using a Deep Ensemble (Simple and scalable predictive uncertainty estimation using deep ensembles by Lakshminarayanan et al. NeurIPS 2017; Bayesian deep learning and a probabilistic perspective of generalization by Wilson and Izmailov NeurIPS 2020) of independently trained models, i.e. $\{\theta_1, \theta_2, ..., \theta_k\} \sim p(\theta|D)$. In this case, the model prediction can be expressed as $p(y|x) = \frac{1}{k}\sum_{i=1}^{k} p(y|x, \theta_k)$.

Specifically, the average predictive entropy of the individual model captures the aleatoric component defined as $H_{aleatoric} = \frac{1}{k}\sum_{k=1}^{k} H[p(y|x, \theta_k)]$, where $H[\cdot]$ denotes the Shannon entropy. The aleatoric uncertainty represents the irreducible noise inherent in the observations. The total uncertainty of the ensemble prediction is given by the entropy of the average predictive distribution as $H_{total} = H[\frac{1}{k}\sum_{k=1}^{k} p(y|x, \theta_k)]$. And the epistemic uncertainty is therefore defined as the difference between the total entropy and the aleatoric uncertainty,

$$H_{epistemic} = H_{total} - H_{aleatoric}$$

Epistemic uncertainty is high when predictions depend strongly on the model parameters θ. If the model is well-trained on sufficient data, knowing actual θ adds little information, so epistemic uncertainty is low.

Fig S1 indicates that for both UKHD internal validation and the external dataset, the aleatoric uncertainty consistently accounts for a large fraction of the total predictive uncertainty, typically above 0.85. This might suggest that the performance of

Hetairos is unlikely to improve much with further data. As the subsampling experiments corroborate, we believe this to be highly misleading. While we expect to see an increase in epistemic uncertainty for the external data, the observed increase in aleatoric uncertainty is not expected as there is no reason to believe that the WSIs from UCL are of lower quality or more morphologically undistinguishable (**Fig. S2**). In addition, an insightful paper from ICML 2025 by Bickford Smith et al. (Rethinking Aleatoric and Epistemic Uncertainty) discusses limitations of the uncertainty decomposition and concludes that the aleatoric-epistemic view is at times not aligned with the actual measures of interest in machine learning.

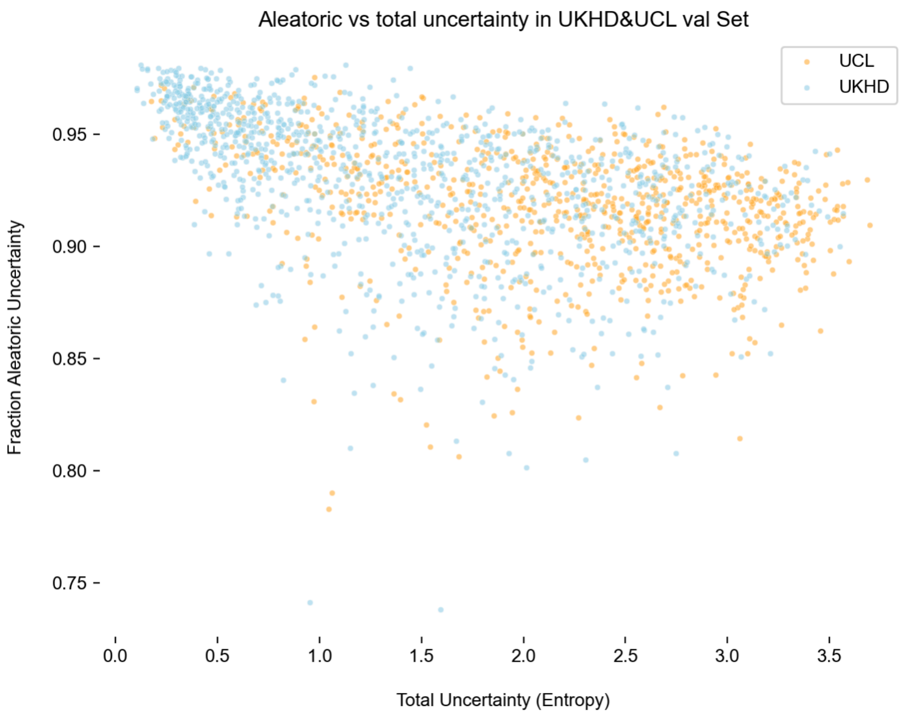

Figure S1. Fraction of aleatoric uncertainty relative to total uncertainty in the UKHD and UCL validation.

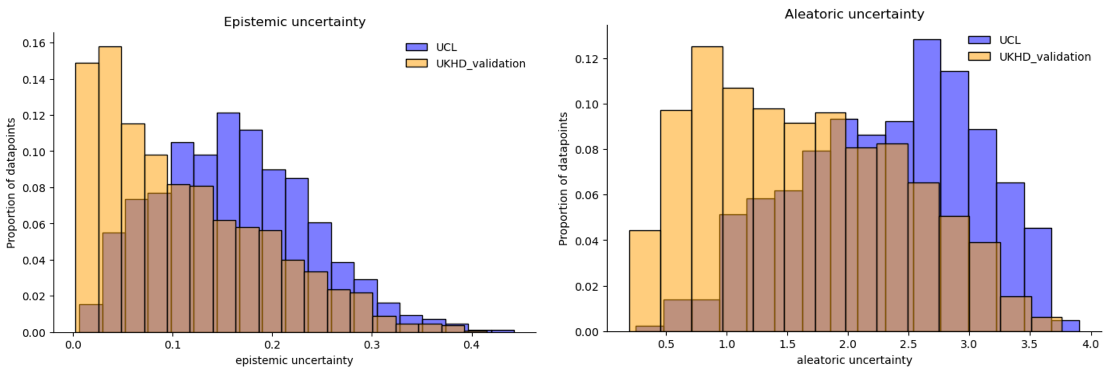

Figure S2. Comparison of epistemic and aleatoric uncertainty distributions in the UKHD and UCL validation cohorts.

Further, we explore the correlation between training sample size and accuracy by conducting a series of subsampling experiments. Specifically, the UKHD cohort is split into five folds, using four folds for training and one fold for testing, yielding five distinct splits. Within each split, training samples for each tumour class were randomly subsampled at rates ranging from 5% to 100%, resulting in 100 models. This systematic design allows assessment of model performance under varying data availability, with the validation accuracies of all models presented in **Extended Data Figure 10**. Accuracy keeps improving with increasing training data sizes. One would expect that a more powerful model such as Hetairos would benefit more strongly from an increase in data size. **Remarkably, the scaling of the accuracy with respect to the training data size is logarithmic**.

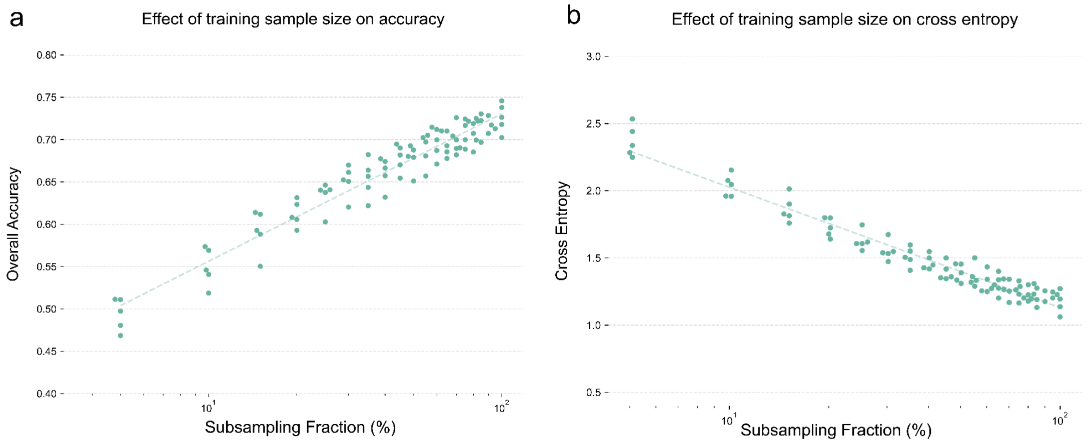

Extended Data Figure 10. a, Scatter plot showing the correlation between training sample size and Hetairos's predicted overall accuracy. **b,** Scatter plot showing the correlation between training sample size and cross entropy values.

The different methylation classes have different scaling behaviours (**Fig. S3**). Some classes do not benefit much from a larger number of data points from their class being in the training data. Adult-type diffuse high-grade glioma, IDH-wildtype, provides a striking example of a class that remains challenging to predict, even with 60 training samples. The model often confuses it with glioblastoma, IDH-wildtype, which is the most prevalent class in the training data and shares similar adult-type, diffuse, and high-grade histopathological features. In contrast, pituitary adenoma represents the opposite extreme: even with only one or two training samples, the model can achieve near-perfect accuracy (**Fig. S4**). This likely reflects its highly distinctive morphology, which sets it apart from all other CNS tumour classes.

Overall, enlarging the training dataset generally enhances the classification performance across most tumour classes. A small subset of entities with indistinct morphological characteristics may show limited sensitivity to dataset expansion. Nonetheless, increasing the dataset's scale and diversity remain a key goal for Hetairos, and the nature of these specific tumours should be further explored in future studies.

We have added the discussion about scaling the training set in the revised manuscript on page 29,

"Both limitations can be resolved with larger and more diverse training cohorts, as also evidenced by our subsampling experiments (**Extended Data Figure 10** and **Supplementary Table 12**), where model accuracy increases logarithmically with training size."

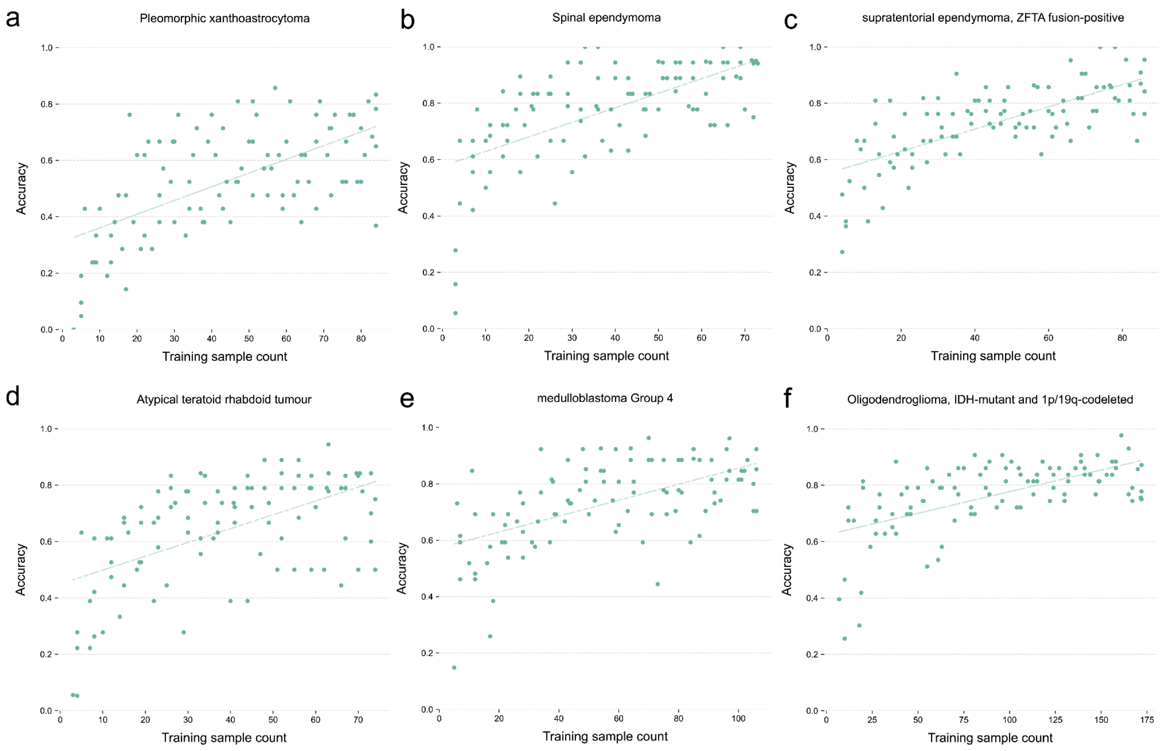

Figure S3. Scatter plot showing the correlation between training sample size and predicted overall accuracy across different tumours.

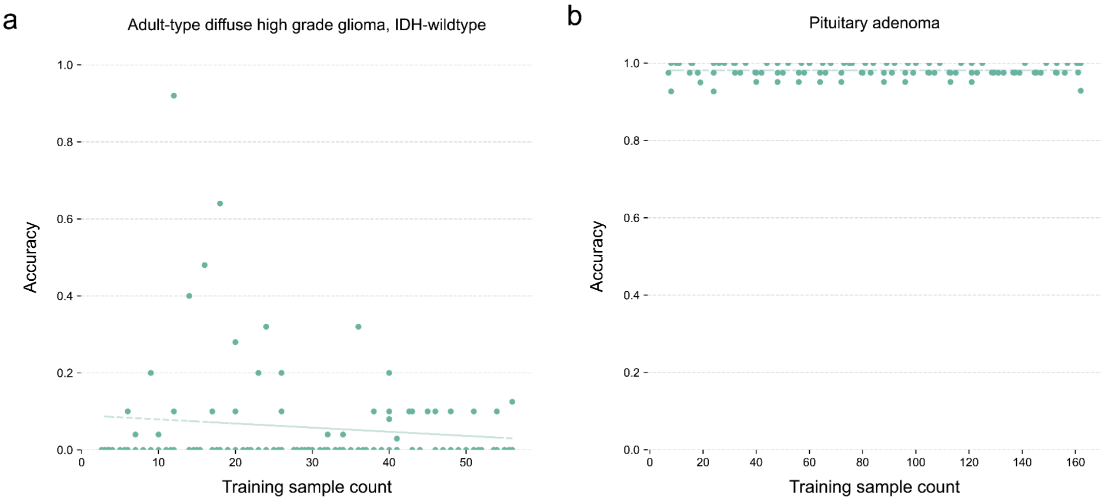

Figure S4. Scatter plot showing the correlation between training sample size and predicted overall accuracy for Adult-type diffuse high-grade glioma, IDH-wildtype and pituitary adenoma.

3 - The authors used a pretrained foundation model for feature extraction. Prov-Gigapath is trained using self-supervision. We have found that self-supervised CPath models are often suboptimal for molecular classification tasks and overfit to morphological features. Did the authors consider fine-tuning the feature extractor using parameter efficient fine tuning (Adapters, LORA, etc.)? With ViT-G this may be challenging, but I would consider trying to better optimize the feature extraction for the task.

We thank the reviewer for the insightful comment regarding the use of the CPath foundation models. We agree that self-supervised histopathology models may primarily capture morphological rather than molecularly informative features.

In our previous experiments, we have indeed observed the potential benefits of fine-tuning foundation models. For example, fine-tuning the CTransPath model on our in-house dataset led to an improvement of approximately **4–5 percent** in classification accuracy. While the Prov-Gigapath model already provides a very strong baseline performance, full fine-tuning of such a large ViT-G model would be computationally heavy and less practical within the current study scope. Therefore, we used it in a frozen feature extraction mode for benchmarking purposes.

Nevertheless, we acknowledge the reviewer's valuable suggestion. And we added a short discussion in the manuscript to highlight the potential of parameter-efficient fine-tuning for large-scale pathology foundation models in Discussion on page 29 as follows:

"Moreover, finetuning foundation models, which are used for feature extraction, with cohort-specific data or through parameter-efficient strategies such as LoRA[39] may further enhance task-specific performance."

Minor comments
1 - Please include an ablation on the performance without age and tumor location. It is helpful and informative to know how much these factors contribute to the prediction. These results should be in extended data figures.

We appreciate this valuable suggestion. We have included an ablation analysis quantifying the contribution of age and tumour location to the prediction as shown in Table S1. Specifically, four models are trained: without age/location, with age only, with location only, and with both age and location, all using identical hyperparameters and evaluated on the UKHD validation set.

According to the results, the model incorporating age and tumour location achieved an overall accuracy **3%** higher than the baseline model. The age only model performed slightly better than that using location alone. As not all test samples have age or location information, we further assessed accuracy on the subset with available information. **Incorporating location alone had negligible impact** compared with the baseline, whereas **age information contributed to an improvement by roughly 2%**. Although the location-only model offered no gain over baseline, **combining location and age yielded best results**, suggesting a complementary effect between two features during training.

Table S1 (frac of samples w age and loc)
ABLATION STUDY ON AGE AND TUMOUR LOCATION.

Model type	Overall acc on UKHD validation	Acc on samples w/ age	Acc on samples w/ location
Model trained w/o age and location	0.718	0.725	0.731
Model trained w/ age	0.729	0.742	0.748
Model trained w/ location	0.720	0.723	0.731
Hetairos (w/ both age and location)	0.750	0.760	0.769

We have added following description in the revised manuscript on pages 6-7 regarding the ablation results:

“Additionally, patient age and the anatomical location of the tumour can be incorporated to enhance model performance (ablation results in **Supplementary Table 1**).”

2 - The paper is very methylation-focused, but this does not appear in the title and is slightly misleading. Consider adding this.

We thank the reviewer for this suggestion. We agree that emphasising the methylation aspect in the title will better reflect the focus of the study. Importantly, our model benefits from the methylation-informed labeling, as our dataset is carefully curated and consistent, in contrast to prior datasets that are often heterogeneous and incorporate multiple versions of the WHO classification.

Therefore, we have decided to modify the title as:

Histology-based AI prediction of central nervous system tumour methylation subtypes.
